# More Hype Than Substance? A Meta-Analysis on Job and Task Rotation

**DOI:** 10.3389/fpsyg.2021.633530

**Published:** 2021-03-25

**Authors:** Lisa Mlekus, Günter W. Maier

**Affiliations:** Department of Psychology, Bielefeld University, Bielefeld, Germany

**Keywords:** job rotation, task rotation, attitudes, health, organizational performance, meta-analysis, work design

## Abstract

Although there exist numerous publications on job and task rotation from various disciplines, there is no consistent evidence of their effectiveness. Drawing on theories from industrial and organizational psychology, knowledge management, ergonomics, and management science, we meta-analytically investigated relationships between job/task rotation and employee attitudes, learning and development, psychological and physical health, and organizational performance. Due to a conceptual overlap and frequent confusion of terminology, we analyzed the design of the rotation (job rotation vs. task rotation) as a possible moderator. The three-level meta-analysis on 56 studies (*N* = 284,086) showed that rotation was significantly associated with job satisfaction (*r* = 0.27), organizational commitment (*r* = 0.16), career success (*r* = 0.31), labor flexibility (*r* = 0.32), general psychological health (*r* = 0.20), stress/burnout (*r* = −0.13), individual performance (*r* = 0.13), and productivity (*r* = 0.13). Positive relationships between rotation and physical health could only be found when rotation was compared to high-intensity work. Task rotation yielded stronger relationships with attitudinal outcomes, job rotation with learning and development, psychological health, and organizational performance outcomes. Further moderator analyses showed that individualism decreased relationships between task rotation and attitudes, and correlations with organizational performance and physical health were stronger for subjective measures. The findings indicate that many expectations toward job and task rotation are not fully supported.

## Introduction

Job and task rotation describe techniques where employees shift periodically and in a planned manner between a range of jobs or tasks within an organization (He et al., 2016; Jones and James, 2018). The first, rather unsystematic appearance of the term *job rotation* dates back to the 1940s and 1950s, when work design methods started to counteract the simplification, specialization, and repetitiveness that dominated the Tayloristic work design of the early twentieth century (Tucker, 1942; Morris, 1956). Since then, rotation has oftentimes been recommended in textbooks and practitioner literature in the fields of industrial and organizational (I/O) psychology (e.g., Jex and Britt, 2014), organizational behavior (e.g., Robbins and Judge, 2017), human resources management (e.g., Armstrong and Taylor, 2017), and engineering (e.g., Kutz, 2014). Despite its widespread use, a closer look at the literature reveals that the label *job rotation* is not used in a consistent way. It describes the rotation either between different jobs (Hsieh and Chao, 2004; Mohsan et al., 2012), between different tasks (Weichel et al., 2010; Jeon et al., 2016), or both (Colombo et al., 2007; Kim et al., 2016). Although job and task rotation are conceptually similar, this impreciseness in terminology could lead to false conclusions. Overall, there are more than 800 publications on job and task rotation from all over the world, and the number of articles as well as citations has been steadily growing (Posthuma et al., 2013; Web of Science, 2021). In the CRANET survey of 2014/15, more than 50% of U.S. organizations reported that they practiced job rotation (Cranet, 2017). They anticipate multiple advantages from rotation: employees with greater satisfaction and motivation due to a reduction of monotony; more skill development due to a greater variety of stimulating work environments; a healthier workforce due to a decrease in monotony and muscle fatigue; and an increase in organizational performance due to greater labor flexibility and a stronger stimulation of organizational learning. Existing studies seem to support these expectations at first glance. In jobs with rotation, they found, for example, greater motivation (*r* = 0.44; Muramatsu et al., 1982; where necessary, values are converted to correlation coefficient *r* for easier comparison) and labor flexibility (*r* = 0.57; Sawhney, 2013), decreased mental fatigue (*r* = −0.32, Jones and James, 2018), a lower incidence of carpal tunnel syndrome (*r* = −0.23; Roquelaure et al., 1997), and increased process innovation performance (*r* = 0.21; Pini and Santangelo, 2005). However, some studies also reported contradicting significant results for motivation (*r* = −0.17; Mohsan et al., 2012), employee adaptability (*r* = −0.41; Zhu et al., 2013), employee energy (*r* = −0.09; Luger et al., 2016*)*, incidence of upper-extremity musculoskeletal disorders (*r* = 0.07; Roquelaure et al., 2009), and innovation performance (*r* = −0.11; Song et al., 2010). Thus, despite much interest in job and task rotation from a variety of disciplines and from researchers and practitioners alike, there are still questions left unanswered: Does rotation really provide the benefits that organizations expect? Is the interchanging use of the terms *job rotation* and *task rotation* justified, or are there differential effects for the interventions? How does the study context affect relationships between rotation and beneficial outcomes? In this manuscript, we present a meta-analytic integration of the relationships between rotation and beneficial outcomes and aim to provide answers to these questions. The participants of the included studies were either employees affected by rotation, managers reporting about rotation in their organization, or student samples in experimental settings. Our aim was to compare great levels of rotation with small levels of rotation (e.g., many job changes vs. few job changes, rotation vs. no rotation) and their relationship with a variety of outcomes (e.g., job satisfaction, career success, stress and burnout, musculoskeletal complaints, and speed of product development). We used the PRISMA reporting guidelines (see Supplementary Material, Supplementary Table 8, for PRISMA checklist).

This manuscript makes several contributions to the literature. First, this is the first meta-analysis and most comprehensive integration of outcomes of job and task rotation. So far, there have been only narrative reviews (e.g., Leider et al., 2015; Padula et al., 2017), and also, these are almost exclusively focused on physical health criteria, such as musculoskeletal complaints or physical strain. Narrative reviews have the limitations that they do not consider measurement error in primary studies, and particular studies might be overweighted or underweighted such that conclusions can be misleading (Schmidt and Hunter, 2015). Moreover, the existing reviews mostly do not cover outcomes from the fields of I/O psychology and management science (e.g., employee development or performance). Second, our meta-analysis contributes to theoretical knowledge about the mechanisms of rotation. We use the interdisciplinary approach to work design of Campion and Thayer (1985) as a guide for possible outcomes of rotation, and complement it with other theories and models from multiple disciplines to explain why rotation might have beneficial effects and under which conditions these effects might increase or decrease. As potential moderating factors, we point out context-related differences regarding the societal culture, investigate differences due to the work intensity in the non-rotation condition, and show to what extent the design of the rotation has an impact on the relationship between rotation and possible beneficial effects. By doing that, we acknowledge conceptual differences between job rotation and task rotation that have been neglected by some previous studies. Third, the meta-analysis provides relevant information for practitioners. The results can give guidance to managers who need to know about the effects of rotation, as well as potential differences between job and task rotation, when considering its implementation. In conclusion, the purpose of this manuscript is to help in understanding the effects of job and task rotation, explain when and where rotation works, and make transparent those areas where we are still lacking knowledge.

## Conceptual Overview of Job Rotation and Task Rotation

Job rotation refers to a lateral transfer of employees within an organization without a change in salary or hierarchy (Campion et al., 1994). It most commonly describes a change between different functions, departments, or units (Dinis and Fronteira, 2015; Le Meunier-Fitzhugh and Massey, 2019). Task rotation also includes a move between job tasks, but on a smaller scale. More specifically, it refers to the alternation between tasks within a job that can require different skills and responsibilities but is not associated with a change to a different function or department (Jeon et al., 2016; Jones and James, 2018). In the past, job and task rotation have not been strictly separated. Some authors defined job rotation as a change between jobs or tasks (e.g., Kim et al., 2016; Comper et al., 2017). Others used the label *job rotation* but actually measured a change of job tasks (e.g., Bao et al., 2016). Then again others used the term *task rotation* to refer to a transfer between functions (e.g., Tsai and Huang, 2020).

The fact that there are no prevailing definitions of job and task rotation could be attributed to the fact that both interventions are based on a change of work settings, and that making a distinction between tasks and jobs is often difficult. Yet, in comparison to task rotation, job rotation refers to more severe job changes. Thus, it probably requires more initial training and a longer time to adjust to the new job, and is more likely to be associated with a change in work environment, colleagues, or supervisors. Additionally, it is likely that job rotation indicates a longer time interval between rotations than task rotation. These arguments are supported by Eriksson and Ortega's (2006) employee learning hypothesis of job rotation. They argued that interfunctional job rotation could be a way to prepare employees for management positions, whereas intrafunctional rotations (i.e., task rotations) are primarily aimed at being able to reallocate employees across different tasks. They also stated that this latter rotation was only efficient when employees already had experience in the tasks and thus did not need much initial training.

Both job rotation and task rotation describe workplace interventions aimed at improving outcomes for employees and the organization. Since research on rotation stems from various disciplines, its outcomes are also multifaceted. In their historical overview of work design research, Parker et al. (2017) identified the interdisciplinary approach of Campion and Thayer (1985) as the starting point of integrative perspectives of work design. Campion and Thayer analyzed work design characteristics from the four disciplines of organizational psychology, human factors, ergonomics, and industrial engineering, and showed that the disciplines are typically aimed at different goals, namely positive employee attitudes (e.g., job satisfaction), reliability (e.g., reduced stress), physical well-being (e.g., few health complaints), and efficiency (e.g., reduced idle time), respectively. To address the multidisciplinarity of rotation research, we investigated in our meta-analysis the relationships between rotation and employee attitudes, psychological health (which Campion and Thayer subsumed under reliability), physical health, organizational performance (which is a broader concept than Campion and Thayer's efficiency), and employee learning and development. Although this last outcome was not a work design goal in Campion and Thayer's approach, more recent publications emphasize its importance in work design research and theory (Parker, 2014, 2017). In the following, we will outline in more detail the theoretical background of attitudinal, developmental, psychological and physical health-related, and organizational outcomes of rotation.

## Rotation and Employee Attitudes

One of the most influential theories of psychological work design, the job characteristics model of Hackman and Oldham (1976), explains why rotation may result in more positive employee attitudes. The authors stated that the five job characteristics of skill variety, task identity, task significance, autonomy, and feedback affect job-related outcomes, such as motivation and satisfaction. The job characteristics model has been complemented by Morgeson and Humphrey (2006) and Humphrey et al. (2007). The authors added knowledge characteristics, social characteristics, and characteristics of the work context. In their meta-analysis (Humphrey et al., 2007), they found evidence for this extended model.

While there already exists cumulative knowledge on single work characteristics (meta-analyses by Fried and Ferris, 1987; Humphrey et al., 2007), there is a unique combination of characteristics that distinguishes jobs with rotation from jobs without rotation. On the one hand, it is likely that the rotation between tasks or jobs increases the perceived variety of tasks, requires a greater variety of skills, and in some cases makes a job more holistic because the tasks or jobs add up to a complete cycle of a work process. Humphrey et al. (2007) found in their meta-analysis positive relationships between these characteristics (task variety, skill variety, and task identity) and positive employee attitudes, such as job satisfaction, internal work motivation, job involvement, and organizational commitment. We assumed that a job that provides a combination of these characteristics, as we expect to be the case in jobs with rotation, is also associated with positive employee attitudes.

On the other hand, jobs with rotation might decrease the experience of autonomy regarding the scheduling of work tasks because employees might be required to follow a fixed rotation roster. In their meta-analysis, Humphrey et al. (2007) investigated the relationships between autonomy and job satisfaction (there were not enough primary studies to investigate other outcomes). They found only significant associations between job satisfaction and other types of autonomy (e.g., work methods autonomy), but not between job satisfaction and work scheduling autonomy. Hence, even a fixed rotation schedule should not affect the positive relationship between rotation and employee attitudes.

*Hypothesis 1*: Rotation is positively associated with the employee attitudes (a) job satisfaction, (b) work motivation, (c) job involvement, and (d) organizational commitment.

## Rotation and Learning and Development

A more recent expansion of the job characteristics model—the work design growth model—was proposed by Parker (2017). This model states that the way work is designed also influences several short-term (e.g., a change in cognition or skills) and long-term learning and development outcomes (e.g., an increase in intellectual flexibility), which had been neglected in previous work design models. Applied to an employment with job or task rotation, it is conceivable that the greater levels of task variety and task identity enhance learning because employees are introduced to new knowledge domains and gain a broader perspective of organizational processes. This notion is supported by a study with 5,800 working participants by Weststar (2009). Here, a change in skill level required to perform a job and a change in work techniques and equipment (both core features of jobs with rotation) were significantly associated with an increase in employees seeking advice from someone knowledgeable with the intention of developing their job skills. Additionally, Antonioli and Della Torre (2016) found in their study of 118 small and medium enterprises that the adoption of job rotation was negatively associated with formal training. The authors interpreted this finding to mean that the investigated companies may adopt job rotation as a substitute for formal learning approaches.

Another explanation is that rotation facilitates the creation of tacit knowledge. Tacit knowledge refers to knowledge that is acquired through experience because it cannot be explicitly verbalized (Nonaka and Takeuchi, 1995). When employees rotate between jobs or tasks, it is more likely that they share their tacit knowledge and learn from each other because they might have more contact with colleagues from other disciplines (Kane et al., 2005). This knowledge acquisition in a variety of jobs or tasks allows employers to deploy their workers more flexibly.

Lastly, the meta-analysis by Humphrey et al. (2007) indicates that rotation might facilitate not only competence development but also career development because they found positive relationships between several rotation-specific work characteristics (i.e., task variety, skill variety, and task identity) and satisfaction with promotion. Thus, we proposed the following hypothesis.

*Hypothesis 2*: Rotation is positively associated with the employee development indicators (a) competence development, (b) career success, and (c) labor flexibility.

## Rotation and Psychological Health

According to an integrative model of psychologically healthy workplaces, employee well-being can be ensured by reducing negative demands and stressors and promoting organizational resources (Kelloway and Day, 2005). Thus, the model suggests changing the objective working conditions, as opposed to addressing individual perceptions and attitudes (Hurrell, 2005).

It can be argued that rotation benefits psychological health because it reduces the job stressors repetitiveness and imbalanced workload. In a review about boredom at work, Loukidou et al. (2009) found that repetitive and monotonous jobs were associated with, for example, psychological distress, depression, and feelings of hostility. Consequently, there are many simulation studies that aim to find an algorithm for job rotation scheduling that diminishes employee boredom (e.g., Bhadury and Radovilsky, 2006; Azizi et al., 2010). Additionally, it is possible that employees' psychological health is positively affected by rotation because the workload is more balanced than in jobs with a single activity, which improves physical health (as described in the following section). Previous studies found a high correlation between physical and psychological health (e.g., Bonzini et al., 2015).

Besides the reduction of these stressors, rotation also provides certain resources. Warr (1999) summarized ten potential environmental determinants of well-being, two of them being variety and opportunities for skill use. As described above, these are assumed to be provided by jobs with rotation. Sevastos et al. (1992) found significant associations between the well-being factors of anxiety-contentment and depression-enthusiasm and the job characteristics of skill variety and task identity. We proposed the following hypothesis.

*Hypothesis 3*: Rotation is (a) positively associated with general psychological health, and (b) negatively associated with stress and burnout.

## Rotation and Physical Health

A model developed by Westgaard and Winkel (1996), based on a review of guidelines for occupational musculoskeletal load, explains why rotation can have an effect on a wide variety of health-related outcomes. The authors state that environmental exposure at work leads to individual reactions in the body, which then cause acute physiological and psychological responses, such as fatigue, change in heart rate, and (dis)comfort. Ultimately, these lead to improved or impaired musculoskeletal health. One important environmental exposure in the workplace proved to be repetitive or monotonous work (Andersen et al., 2002). Increased repetitiveness means that one particular body region is continuously stressed, and the affected internal structures have little opportunity to recover (Luger et al., 2014). As a relief, employees could either have more rest breaks or change between tasks that stress different body regions, and thus engage in task rotation (Luger et al., 2014).

Previous literature reviews on the effects of task rotation on physical health found ambiguous results. On the one hand, reviews about task rotation and shoulder fatigue (Luger et al., 2014), muscular activity variability (Rodriguez and Barrero, 2017), or work-related musculoskeletal disorders and sick leave (Padula et al., 2017) reported (weak) positive effects of task rotation on physical health. On the other hand, reviews about task rotation and musculoskeletal complaints and physical workload (Leider et al., 2015) or upper limb muscle fatigue (Santos et al., 2016) found inconsistent effects across studies. The authors discussed several explanations: First, the overall effect of rotation might have been canceled out because employees who normally performed high-intensity work benefitted from rotation, whereas employees who normally perform low-intensity work experienced a disadvantage due to the introduction of rotation (Luger et al., 2014; Leider et al., 2015). Second, it is possible that the tasks within a rotation cycle did not stress different body regions so that the expected beneficial effects could not unfold. Leider et al. (2015) described, for example, a study where the employees had to work above shoulder level and do repetitive hand movements for an extended time both before and after the introduction of rotation. Mathiassen (2006) noted that there are currently no appropriate metrics to determine the diversity of exposed body regions.

To account for the previous ambiguous results, we assumed the following hypothesis.

*Hypothesis 4*: The associations between rotation and physical health outcomes are moderated by the work intensity of the reference group. If the reference group performs high-intensity work, there is a (a) positive association with general physical health, and a negative association with (b) musculoskeletal complaints and (c) physical workload. If the reference group performs low-intensity work, there is a (d) negative association with general physical health, and a positive association with (e) musculoskeletal complaints and (f) physical workload.

## Rotation and Organizational Performance

We drew on resource-based theory to explain why job and task rotation may affect organizational performance. The theory states that the major determinant of an organization's success is its internal resources, one of them being human capital resources (e.g., experience of managers and workers; Barney, 1991; Barney et al., 2011). Ensuing from human capital resources in resource-based theory, there are two explanatory approaches for the effect of rotation on organizational performance: workforce flexibility and organizational learning.

First, workforce flexibility ensues from rotation because, as described above, rotation fosters employee development, and thus proficiency in a variety of jobs and tasks. This labor flexibility helps to avoid bottlenecks, reduce idle time, and achieve a shorter lead time. All of these contribute to an enhanced financial performance of the organization (Bhattacharya et al., 2005; Beltrán-Martín et al., 2008). Additionally, the work characteristics of task variety and task identity have also been found to be positively related to individual, subjective performance (Humphrey et al., 2007).

Second, organizations can use rotation as a method to convert individual resources (i.e., employee knowledge and skills) into organizational knowledge, a process called *organizational learning* (Maier et al., 2001; Basten and Haamann, 2018). This process reduces employee turnover and is critical to an organization's innovative capabilities, which, in turn, should translate into organizational performance (Egan et al., 2004; Jiménez-Jiménez and Sanz-Valle, 2011). One important component of organizational learning theories is the transfer of knowledge among employees (Nonaka, 1994; Argote, 2013). This knowledge sharing should be facilitated by rotation activities: Studies on cross-functional teams found that job rotation was associated with increased communication between functions, more involvement in cross-functional activities, and more congruent goals across functions (Hauptman and Hirji, 1999; Xie et al., 2003). Thus, rotation enables a tighter network within the organization (Jansen et al., 2005). These factors can contribute to faster processes, such as product development, greater productivity, and increased innovative capabilities. Thus, we proposed the following hypothesis.

*Hypothesis 5*: Rotation is positively associated with the organizational performance indicators (a) individual performance, (b), productivity, (c) speed of product development, (d) innovativeness, and (e) financial performance, and negatively associated with (f) turnover (intention).

## Potential Moderators of Rotation Outcomes

As described above, previous studies have often confused task rotation with job rotation (or vice versa). Thus, it is possible that ambiguous results from primary studies can be explained by the concrete design of a rotation intervention, which is either a job rotation or a task rotation. Based on the theoretical arguments presented above, one can assume that for some outcomes, the relationships with rotation are stronger for job rotation than for task rotation and conversely for other outcomes. As regards employee attitudes, we expected stronger relationships for task rotation than for job rotation. Task rotation implies a more frequent change between activities so that the perceived task variety and skill variety, which are both associated with positive employee attitudes, should be greater (Humphrey et al., 2007). Additionally, job rotation is often associated with a change to a different workplace, which can result in a lack of social support because employees will have new colleagues. Meta-analytic results indicate that a lack of social support is associated with less positive employee attitudes (Humphrey et al., 2007). In the case of learning and development, it is likely that employees gain a broader perspective from job rotation than from task rotation because they experience more diverse work environments. These are more likely to stimulate learning and growth (Parker, 2017). Based on our reasoning for psychological health, the relationship should be stronger for task rotation than for job rotation. As described above, task rotation is more likely to provide the resource of variety, which was found to be related to less depression and anxiety (Sevastos et al., 1992). Additionally, task rotation is potentially more suitable to reduce the stressor of an imbalanced workload, which should indirectly affect psychological health (Bonzini et al., 2015). With regard to physical health, we expected stronger relationships with task rotation than with job rotation (when compared to high-intensity work) because the recovery of specific strained body parts can be best achieved when the alternation between work activities occurs quite frequently (Mathiassen, 2006). Regarding organizational performance, we believed that job rotation would result in stronger relationships because it more often includes a change to another department. This contributes firstly to a broader picture of the organization and consequently more workforce flexibility (Parker, 2017), and secondly to organizational learning because it encourages more interdepartmental knowledge sharing (Hauptman and Hirji, 1999). We proposed the following hypothesis.

*Hypothesis 6*: The relationship between rotation and (a) employee attitudes, (b) learning and development, (c) psychological health, (d) physical health, and (e) organizational performance is moderated by the concrete design of the rotation (job rotation vs. task rotation).

As another potential moderator, we investigated the context of the primary studies. As Johns (2006) pointed out, it is important to always interpret study results in the light of situational factors that might affect the occurrence of behavior and the relationship between variables. We expected that the collectivism/individualism of the societal culture would have an influence on the relationship between rotation and attitudes. In individualistic cultures, people tend to view themselves as independent individuals. Employees are thus more likely to strive for individual goals and pursue individual interests. In contrast, employees from collectivistic cultures see themselves as part of a collective (e.g., their organization), are motivated by the collective's norms, and are willing to give the collective's goals a higher priority than their own (Triandis, 1995). Task rotation could be more strongly related to positive employee attitudes in collectivistic cultures because it puts an emphasis on the collective's goal by diminishing job specialization and making employees more interchangeable (Fægri et al., 2010). Employees from individualistic cultures, however, might feel that their individual contributions at work cannot be identified in the context of task rotation, which might result in less positive employee attitudes. In regard to the adoption of job rotation it is likely that it is more beneficial for employee attitudes in individualistic cultures than in collectivistic cultures. Job rotation helps employees broaden their skill set and gain a deeper understanding of business operations (Eriksson and Ortega, 2006). As this could ultimately be beneficial for their individual career advancement, the possibility of participating in job rotation might be perceived as a privilege, which results in more favorable attitudes. These individual-oriented goals are theorized to be less relevant for employees from collectivistic cultures (Triandis, 1995).

*Hypothesis 7*: The individualism/collectivism value of the societal culture moderates the relationship between rotation and employee attitudes, based on the concrete design of the rotation. As the societal culture becomes more individualistic, the positive relationships will (a) decrease in the case of task rotation and (b) increase in the case of job rotation.

In addition to the theoretically derived potential moderators, we also addressed a practically relevant aspect that could affect the relationships between rotation and its outcomes: We investigated whether there were any differences depending on whether the outcome was measured subjectively or objectively. Especially from an organization's point of view, objective success indicators are highly relevant because they are believed to be the most accurate representation of the real world and therefore guide future strategic decisions (Andrews et al., 2006). Although often used interchangeably, meta-analytic studies suggest that subjective and objective organizational performance measures are only weakly correlated (e.g., Bommer et al., 1995).

*Research Question*: Are there any differences in the strength of the relationship between rotation and its outcomes based on whether the outcome was measured subjectively or objectively?

## Method

The data underlying the present meta-analysis are openly available in Open Science Framework (OSF^1^).

### Literature Search and Inclusion Criteria

We conducted a variety of search strategies to identify empirical studies published before February 2021. First, we conducted a search in the online databases PsycINFO, PSYNDEX, Education Source, Web of Science, EconLit, and Medline using the search term “*job rotation” OR “task rotation.”* Second, we conducted a manual search of all conference programs that were available online of the Society for Industrial and Organizational Psychology (1998–2020), Academy of Management (1954–2020), European Association of Work and Organizational Psychology (2007–2019), and International Ergonomics Association (2015–2018) conferences. Third, we manually searched major journals from the fields of I/O psychology, management, health, and ergonomics, including the *Journal of Organizational Behavior, Journal of Applied Psychology, Personnel Psychology, Organization Science, Journal of Occupational Health Psychology, Applied Ergonomics, Health Psychology*, and *Work & Stress*. Fourth, we examined the reference lists from previous literature reviews on job rotation and pertinent topics (e.g., Leider et al., 2015; Padula et al., 2017; Basten and Haamann, 2018). Lastly, we conducted a manual search of the reference lists of all included articles. In an effort to obtain more gray literature, we complemented these search strategies with further approaches. More specifically, we posted a call for unpublished data in the Calls and Announcements section on the website of the Society for Industrial and Organizational Psychology^2^ and via the mailing list of the German Psychological Society. As the European Association for Work and Organizational Psychology does not have a mailing list or announcements section on their website, we shared our call for unpublished data in the corresponding LinkedIn group.^3^ Additionally, we contacted all authors of primary studies that we had identified thus far and asked whether they had further unpublished data that we could include.

We included all studies that reported a sample size and an effect size, or enough information to calculate it, and examined a unique sample that had not been included in this meta-analysis already. In line with past meta-analyses, we only included outcomes of job and task rotation when they were represented in at least three independent samples (cf., Berry et al., 2007; Eby et al., 2008; Kleine et al., 2019). We included experimental studies, quasi-experimental studies, and correlational studies in all languages. In studies in a language other than English, German, or French, we retrieved the relevant information using Google Translate.^4^ Due to the recommendation by Roth et al. (2018), we excluded studies that only reported regression weights and where we could not obtain zero-order correlations from the authors.

### Coding Procedures

For the coding of the included studies, we compiled a manual that described the coding procedure, including all relevant coding decisions. The first author coded all studies, and another I/O psychologist familiar with the coding procedure coded a randomly selected 30% of the studies. We assessed the interrater agreement for categorical variables with Cohen's kappa, and the interrater reliability for continuous variables with intraclass coefficients (ICC 2,1) after all studies were coded. The kappa coefficients ranged from 0.76 (level of operationalization) to 1 (e.g., nationality), and the ICC from 0.99 (mean age) to 1 (e.g., sample size). Overall, these analyses showed good to very good interrater agreement and reliability. The discrepancies among the coders were then resolved by discussion between the coders, and the first author re-evaluated the coding decisions of the single-handedly coded studies based on the aspects that were discussed most frequently.

The effect size metric was the correlation coefficient Pearson's *r*. We coded either *r* directly, another effect size that could be converted to *r* (e.g., odds ratio for the incidence of low back pain), or the necessary information to calculate an effect size that could be converted to *r* (e.g., means and standard deviations). For the conversion, we used formulae by Borenstein et al. (2009). We included studies with a between-subjects design as well as those with a within-subjects design. Borenstein et al. (2009) argue that it is legitimate to combine studies with different designs as long as they aim to answer the same question. When studies used a within-subjects design, we first calculated Cohen's *d* using the formula provided by Cheung (2015a), which accounts for the dependency between pre- and post-values by including the intercorrelation, and then converted it to *r*. When studies used two independent groups with repeated measures, we used the formula provided by Lipsey and Wilson (2001). In two cases (Kuijer et al., 2005; Comper et al., 2017), we could not obtain the intercorrelation for the within-person values, so we only coded the between-person effect size for the post-values.

#### Coding of Methodological Factors and Study Characteristics

Publication status of the study was coded as a dummy variable (peer-reviewed publication vs. unpublished). For the study design, we coded whether rotation and the corresponding outcome were assessed concurrently or if the outcome was assessed after rotation. Thus, the binary variable had the two categories cross-sectional and time-lagged. Other design factors that we coded were the study setting (laboratory vs. field) and whether the study used a within- or between-subjects design. A within-subjects design meant that participants of the primary study were their own control group because they were assessed before and after the rotation intervention. A between-subjects design meant that participants with and without rotation (or with varying degrees of rotation) were compared with each other. Additionally, we coded the study rigor using an ordinal variable with the categories experiment (greatest rigor), quasi-experimental study, and correlational study (lowest rigor). As there were only few studies with an experimental or quasi-experimental design, we later combined these categories in our calculations.

#### Coding of Outcomes

For some outcomes, we decided to create synthetic construct groupings because primary studies reported very similar, conceptually overlapping constructs. We analyzed the operationalizations of each construct and logically combined semantically similar constructs. A table with all synthetic constructs and the underlying operationalizations can be found in the Supplementary Table 1.

#### Coding of Moderators

In terms of the concrete design of the rotation, we created a categorical variable with the groups job rotation, task rotation, and both. The coding was based on the measurement of rotation (not on the definition the primary authors provided). An example description that indicated job rotation is “any change in job title or department that did not coincide with an increase in salary” (Campion et al., 1994, p. 1525), an example that indicated task rotation is “a dichotomous question asking whether an employee's job involves rotating tasks between the employee and colleagues” (Avgoustaki, 2016, p. 663), and an example of both is “do operators rotate across jobs or tasks on the line?” (Colombo et al., 2007, p. 1045). To investigate the relationships between rotation and physical health outcomes, we coded whether the control group performed tasks with a higher work intensity or lower work intensity. For the cultural moderator, we used the dimension *individualism/collectivism* by Hofstede (2001). Every study that provided information on the country of data collection was assigned the individualism/collectivism index for this country. The values ranged from 1 to 100, with higher scores indicating greater levels of individualism. To address our research question, we also coded whether the outcome measure was a subjective (e.g., a self-rating questionnaire) or an objective (e.g., company data) measure.

### Meta-Analytic Procedure

Most of our included studies reported more than one effect size. These effect sizes are usually dependent, which is why traditional meta-analytic procedures (e.g., Schmidt and Hunter, 2015) require the meta-analyst to include only a single effect size per study. Common strategies to accomplish this are, for example, calculating composites or selecting one effect size per sample. These strategies, however, result in an underestimation of heterogeneity and a loss of information (Cheung and Chan, 2004; Cheung, 2014). Hence, we decided to perform a three-level meta-analysis, which accounts for dependencies of effect sizes (Van den Noortgate et al., 2013; Cheung, 2015a).

Traditional meta-analytic procedures can be regarded as two-level models, with participants at Level 1 and studies at Level 2. That means that effect sizes vary due to two types of variance: sampling variance and between-study variance. With the use of a three-level model, it is possible to consider a third source of variance: within-study variance, which can result, for example, from the use of several measures for the same criterion, or from the measurement of various criteria in one study. The resulting three levels were participants at Level 1, effect sizes within studies at Level 2, and studies at Level 3.

We calculated the sampling variance of the effect sizes (Level 1 variance) using formulae provided by Cheung (2015a, Chapter 3) and Borenstein et al. (2009, Chapter 7). To calculate the mean effect sizes across studies (*r*) and the heterogeneity of effect sizes τ^2^ within studies (Level 2) and between studies (Level 3), we used the metaSEM package (Version 1.2.5; Cheung, 2015b) for R (Version 4.0.1; R Core Team, 2020). The package calculates significance (*p*-values) and 95% confidence intervals based on Wald approximations (*Z*).

## Results

### Study Characteristics

**Figure 1:**
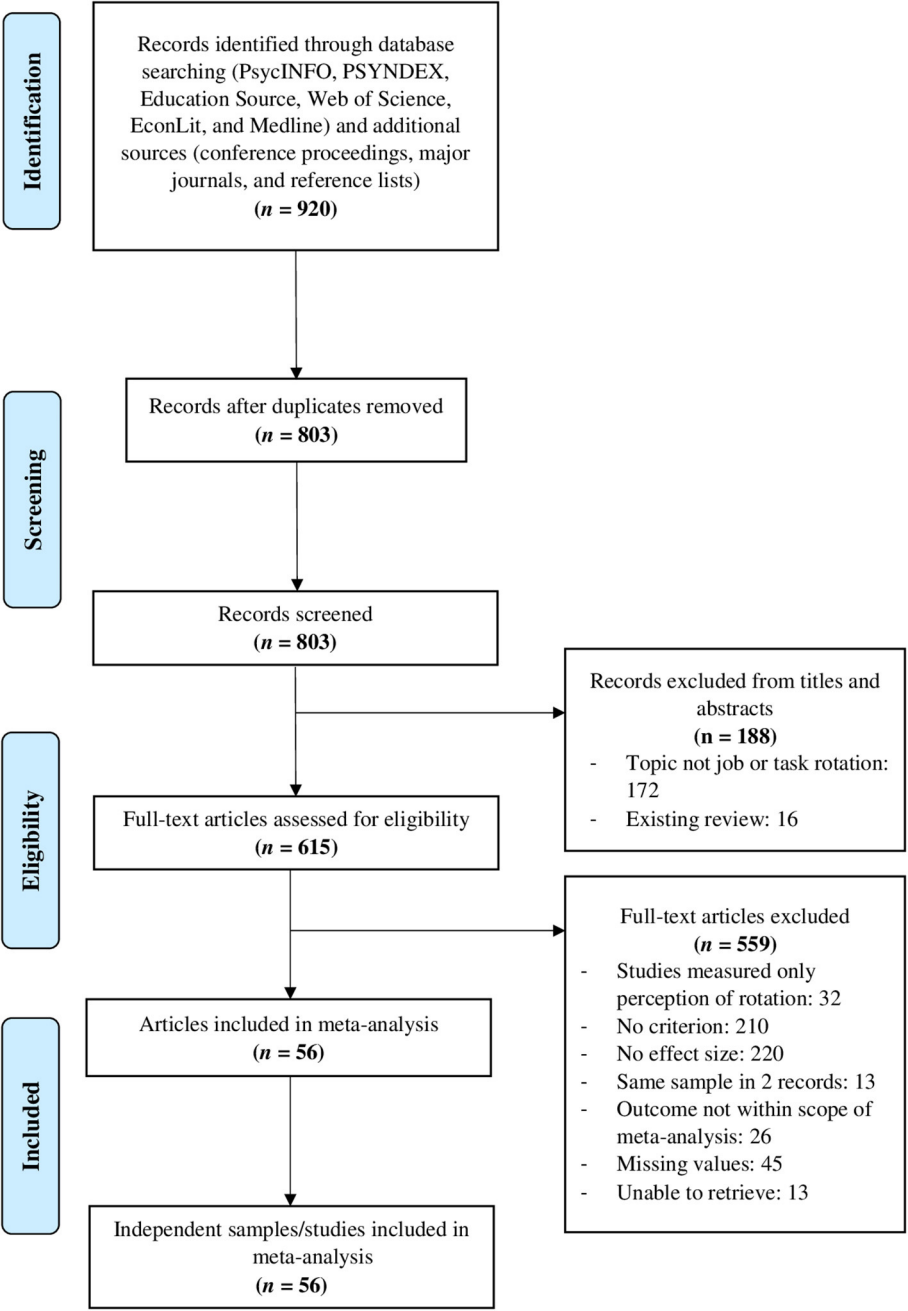
Flow chart with reasons for article exclusions.

Our literature search yielded 803 hits (excluding duplicates). After excluding studies according to our predefined criteria, the analyses are based on a total of 56 studies, 253 effect sizes, and 284,086 participants, reported in 56 articles (see Figure 1 for a flow chart depicting reasons for article exclusions). An overview of all included studies with the investigated constructs, operationalizations, and the respective effect sizes can be found in Supplementary Table 9.

Overall, 53 articles were peer-reviewed publications and three were unpublished studies (one working paper, one news article without peer review, and one unpublished data set). The primary studies were carried out between 1982 and 2020. We could not include any earlier articles because these were either not empirical studies or they were qualitative and thus did not report an effect size. Most studies were conducted in Europe (*k* = 24), followed by North America and Asia (both *k* = 13), mixed samples (*k* = 3), South America, Australia, and Africa (all *k* =1). On average, the samples had a mean age of 34.73 years (*SD* = 6.30; *Min* = 22; *Max* = 42) and were 47.64% female. The majority of the samples were employees (*k* = 37), followed by managers (*k* = 16), and students (*k* = 3). Most studies had a correlational design (*k* = 46), eight studies were experiments, and two studies used a quasi-experimental design.

### Relationships Between Rotation and Employee-Related and Organizational Outcomes

To test the relationships between rotation and employee-related and organizational outcomes, we computed a mixed-effects three-level meta-analysis that included the type of outcome as a covariate (see Table 1). As all outcomes were included in this analysis, we reverse-coded the effect sizes of those outcomes that indicate a negative effect: stress and burnout, musculoskeletal complaints, physical workload, and turnover (intention). Thus, positive values in these outcomes indicate a reduced level of the respective outcome. The results showed significant correlations between rotation and job satisfaction (*r* = 0.27, *p* < 0.001), organizational commitment (*r* = 0.16, *p* = 0.02), career success (*r* = 0.31, *p* = 0.002), labor flexibility (*r* = 0.32, *p* = 0.004), general psychological health (*r* = 0.20, *p* = 0.01), stress and burnout (*r* = 0.13, *p* = 0.02), individual performance (*r* = 0.13, *p* = 0.02), and productivity (*r* = 0.13, *p* = 0.02). These correlations exceed in magnitude between 27 and 75% of effect sizes reported in the human resources and organizational behavior literatures (Paterson et al., 2016). Thus, the results supported our Hypotheses 1a, 1d, 2b, 2c, 3a, 3b, 5a, and 5b. They did not support our Hypotheses 1b (work motivation), 1c (job involvement), 2a (competence development), 5c (speed of product development), 5d (innovativeness), 5e (financial performance), and 5f (turnover).

**Table 1:** Relationships between job and task rotation and outcomes.

**Outcomes**	***k***	***n*_**es**_**	**Estimate**	***SE***	**CI 95%**		***Z***	***p***	***R*^**2**^**
					**LL**	**UL**			
**Attitudes**									
Job satisfaction	8	21	0.27	0.05	0.17	0.37	5.39	<0.001	
Work motivation	6	6	0.12	0.08	−0.04	0.28	1.44	0.15	
Job involvement	6	6	0.10	0.08	−0.05	0.26	1.28	0.20	
Organizational commitment	7	8	0.16	0.07	0.03	0.29	2.36	0.02	
**Learning and development**									
Competence development	4	7	0.13	0.08	−0.02	0.30	1.63	0.10	
Career success	3	5	0.31	0.10	0.11	0.51	3.07	0.002	
Labor flexibility	3	4	0.32	0.11	0.10	0.55	2.85	0.004	
**Psychological health**									
General psychological health	5	7	0.20	0.08	0.05	0.36	2.55	0.01	
Stress and burnout^a^	10	17	0.13	0.06	0.02	0.24	2.35	0.02	
**Physical health**									
General physical health	6	8	0.12	0.07	−0.02	0.26	1.63	0.10	
Musculoskeletal complaints^a^	12	72	0.08	0.04	−0.00	0.17	1.91	0.06	
Physical workload^a^	6	27	0.13	0.07	−0.00	0.26	1.95	0.05	
**Organizational performance**									
Individual performance	10	18	0.13	0.06	0.02	0.24	2.31	0.02	
Productivity	3	24	0.13	0.06	0.03	0.24	2.41	0.02	
Speed of product development	3	3	0.17	0.13	−0.09	0.42	1.30	0.19	
Innovativeness	5	8	0.12	0.09	−0.06	0.30	1.58	0.11	
Financial performance	6	7	0.13	0.08	−0.03	0.30	1.58	0.11	
Turnover (intention)^a^	5	5	0.12	0.09	−0.06	0.29	1.28	0.20	
$\tau_{\left(Level\;2\right)}^{2}$			0.02	0.00	0.02	0.03	7.39	<0.001	0.16
$\tau_{\left(Level\;3\right)}^{2}$			0.03	0.01	0.01	0.04	3.42	<0.001	0.00

k, number of independent samples; n_es_, number of effect sizes; CI, confidence interval; LL, lower level; UL, upper level; Z, Wald approximation; R^2^, estimated heterogeneity at Level 2 and Level 3 that is explained by the outcomes; $\tau_{\left(Level\;2\right)}^{2}$, heterogeneity of effect sizes within studies; $\tau_{\left(Level\;3\right)}^{2}$, heterogeneity of effect sizes between studies.

a *Reverse-coded; high values indicate low levels of stress and burnout, musculoskeletal complaints, physical workload, or turnover (intention)*.

Regarding the relationships between rotation and physical health, we had not assumed a general positive or negative relationship. Instead, we expected differences due to the work intensity of the reference group. Most studies did not specify which tasks were performed by the reference group, or work intensity was similar in the rotation and the non-rotation condition. Also, there was only one study that reported a comparison between rotation and high work intensity and investigated an effect of job rotation on general physical health (Han et al., 2020, *r* = 0.17). Thus, we could not test Hypotheses 4a and 4d. To test the other hypotheses on physical health, we investigated the subsample of rotation vs. low work intensity and the subsample of rotation vs. high work intensity (see Table 2). In line with our hypotheses, when the reference group performed high-intensity work, there were negative relationships between rotation and musculoskeletal complaints (*r* = −0.38, *p* = 0.003) and physical workload (*r* = −0.32, *p* = 0.01). These results support Hypotheses 4b and 4c. When the reference group performed low-intensity work, there were positive relationships between rotation and musculoskeletal complaints (*r* = 0.16, *p* = 0.06) and physical workload (*r* = 0.20, *p* = 0.07), but they were smaller and non-significant. Thus, Hypotheses 4e and 4f could not be supported.

**Table 2:** Relationships between job and task rotation and physical health outcomes subdivided according to work intensity of reference group.

**Outcomes**	***k***	***n*_**es**_**	**Estimate**	***SE***	**CI 95%**		***Z***	***p***	***R*^**2**^**
					**LL**	**UL**			
**Subsample: rotation vs. low work intensity**									
Musculoskeletal complaints	3	10	0.16	0.09	−0.01	0.33	1.86	0.06	
Physical workload	3	6	0.20	0.11	−0.01	0.41	1.84	0.07	
$\tau_{\left(Level\;2\right)}^{2}$			0.03	0.02	−0.01	0.07	1.35	0.18	0.02
$\tau_{\left(Level\;3\right)}^{2}$			0.00	0.02	−0.05	0.05	0	1.00	0.00
**Subsample: rotation vs. high work intensity**									
Musculoskeletal complaints	3	10	−0.38	0.13	−0.63	−0.13	−3.01	0.003	
Physical workload	3	6	−0.32	0.12	−0.56	−0.08	−2.57	0.01	
$\tau_{\left(Level\;2\right)}^{2}$			0.02	0.01	−0.00	0.05	1.74	0.08	0.12
$\tau_{\left(Level\;3\right)}^{2}$			0.05	0.04	−0.03	0.12	1.22	0.22	0.28

*k, number of independent samples; n_es_, number of effect sizes; CI, confidence interval; LL, lower level; UL, upper level; Z, Wald approximation; R^2^, estimated heterogeneity at Level 2 and Level 3 that is explained by the outcomes; $\tau_{\left(Level\;2\right)}^{2}$, heterogeneity of effect sizes within studies; $\tau_{\left(Level\;3\right)}^{2}$, heterogeneity of effect sizes between studies*.

### Differences Between Job Rotation and Task Rotation

To investigate whether the concrete design of the rotation affected the relationships between rotation and employee attitudes, learning and development, psychological health, physical health, and organizational performance, we conducted analyses for each outcome category and included the intervention (job rotation vs. task rotation) as a covariate. There was only one study with one effect size that investigated the relationship between job rotation and physical health outcomes. Therefore, we could not test Hypothesis 6d. The results for the other outcome categories are presented in Table 3. As we investigated the overall outcome categories, we again used the reverse-coded effect sizes for stress and burnout and turnover (intention). As expected, the relationship between rotation and employee attitudes was stronger in the case of task rotation (*r* = 0.10, *p* = 0.03) than in the case of job rotation (*r* = −0.00, *p* = 0.97). The difference was, however, non-significant (*r*_Diff_ = 0.11, *p* = 0.23). Also as expected, the relationship between rotation and learning and development was stronger when the intervention was job rotation (*r* = 0.21, *p* = 0.10) than when it was task rotation (*r* = 0.09, *p* = 0.48). Again, the difference was non-significant (*r*_Diff_ = −0.12, *p* = 0.51). Contrary to our expectations, the relationship between rotation and psychological health was stronger for job rotation (*r* = 0.20, *p* = 0.005) than for task rotation (*r* = 0.14, *p* = 0.01). This difference was also not significant (*r*_Diff_ = −0.05, *p* = 0.54). Lastly, in line with our expectations, the relationship between rotation and organizational performance was stronger in the case of job rotation (*r* = 0.12, *p* = 0.002) than in the case of task rotation (*r* = 0.03, *p* = 0.26). This difference was also not significant (*r*_Diff_ = −0.09, *p* = 0.07). In conclusion, the results indicated slight differences between job rotation and task rotation, which were mostly in line with our expectations. As none of these differences were statistically significant, we had to reject Hypotheses 6a–c and 6e.

**Table 3:** Results of moderated meta-analysis that compares job rotation with task rotation for different outcome categories.

**Intervention**	***k***	***n*_**es**_**	**Estimate**	***SE***	**CI 95%**		***Z***	***p***	***R*^**2**^**
					**LL**	**UL**			
**Subsample: attitudes**									
Job rotation	4	7	−0.00	0.07	−0.15	0.14	−0.04	0.97	
Task rotation	9	24	0.10	0.05	0.01	0.20	2.19	0.03	
$\tau_{\left(Level\;2\right)}^{2}$			0.01	0.00	0.00	0.02	2.26	0.02	0.02
$\tau_{\left(Level\;3\right)}^{2}$			0.01	0.01	−0.00	0.03	1.66	0.10	0.12
**Subsample: learning and development**									
Job rotation	4	11	0.21	0.13	−0.04	0.45	1.65	0.10	
Task rotation	4	4	0.09	0.13	−0.16	0.34	0.71	0.48	
$\tau_{\left(Level\;2\right)}^{2}$			0.01	0.01	−0.00	0.02	1.27	0.20	0.00
$\tau_{\left(Level\;3\right)}^{2}$			0.05	0.03	−0.02	0.12	1.51	0.13	0.10
**Subsample: psychological health**									
Job rotation	4	9	0.20	0.07	0.06	0.33	2.82	0.005	
Task rotation	7	12	0.14	0.06	0.03	0.25	2.57	0.01	
$\tau_{\left(Level\;2\right)}^{2}$			0.00	–	–	–	–	–	0.00
$\tau_{\left(Level\;3\right)}^{2}$			0.02	0.01	−0.00	0.03	1.89	0.06	0.07
**Subsample: organizational performance**									
Job rotation	15	19	0.12	0.04	0.04	0.19	3.08	0.002	
Task rotation	8	35	0.03	0.03	−0.02	0.09	1.13	0.26	
$\tau_{\left(Level\;2\right)}^{2}$			0.02	0.01	0.01	0.04	4.29	<0.001	0.06
$\tau_{\left(Level\;3\right)}^{2}$			0.00	–	–	–	–	–	0.00

*k, number of independent samples; n_es_, number of effect sizes; CI, confidence interval; LL, lower level; UL, upper level; Z, Wald approximation; R^2^, estimated heterogeneity at Level 2 and Level 3 that is explained by the intervention; $\tau_{\left(Level\;2\right)}^{2}$, heterogeneity of effect sizes within studies; $\tau_{\left(Level\;3\right)}^{2}$, heterogeneity of effect sizes between studies*.

### Differences Due to Societal Culture

To examine whether collectivism/individualism affected the relationship between rotation and employee attitudes, depending on the concrete design of the rotation, we created subsamples for task rotation and job rotation and added the collectivism/individualism value as a continuous covariate in both subsamples. In the task rotation subsample, with greater levels of individualism, the relationship between rotation and attitudes decreased significantly (*B* = −0.004, *p* = 0.003). Thus, the results supported Hypothesis 7a. In the job rotation subsample, with greater levels of individualism, the relationship between rotation and attitudes increased, however not significantly (*B* = 0.00, *p* = 0.80). Thus, the results did not support Hypothesis 7b.

### Differences Between Subjective and Objective Outcome Measures

To investigate whether there were differences between subjective and objective outcome measures, we conducted analyses for each outcome category and included the measurement type (subjective vs. objective) as a covariate. The only outcome categories that contained any objective outcome measures were physical health and organizational performance. As we had found that the work intensity of the reference group affected the results, we excluded effect sizes that compared rotation to low-intensity work in this analysis. The results (see Table 4) showed that for both outcome categories, the relationship between rotation and subjective outcome measures was stronger (physical health: *r* = 0.21, *p* < 0.001; organizational performance: *r* = 0.18, *p* < 0.001) than between rotation and objective outcome measures (physical health: *r* = 0.07, *p* = 0.23; organizational performance: *r* = 0.01, *p* = 0.88). The difference was significant in both cases (physical health: *r*_Diff_ = −0.14, *p* < 0.001; organizational performance: *r*_Diff_ = −0.19, *p* < 0.001).

**Table 4:** Results of moderated meta-analysis that compares subjective with objective outcome measures for different outcome categories.

**Measure**	***k***	***n*_**es**_**	**Estimate**	***SE***	**CI 95%**		***Z***	***p***	***R*^**2**^**
					**LL**	**UL**			
**Subsample: physical health**									
Subjective	13	41	0.21	0.06	0.09	0.32	3.53	<0.001	
Objective	11	50	0.07	0.06	−0.05	0.19	1.19	0.23	
$\tau_{\left(Level\;2\right)}^{2}$			0.02	0.00	0.01	0.03	4.84	<0.001	0.17
$\tau_{\left(Level\;3\right)}^{2}$			0.04	0.02	0.01	0.07	2.39	0.02	0.00
**Subsample: organizational performance**									
Subjective	20	34	0.18	0.03	0.12	0.25	5.50	<0.001	
Objective	11	31	0.01	0.03	−0.07	0.06	−0.15	0.88	
$\tau_{\left(Level\;2\right)}^{2}$			0.02	0.01	0.01	0.03	3.75	<0.001	0.29
$\tau_{\left(Level\;3\right)}^{2}$			0.00	0.00	−0.01	0.01	0.55	0.58	0.16

*k, number of independent samples; n_es_, number of effect sizes; CI, confidence interval; LL, lower level; UL, upper level; Z, Wald approximation; R^2^, estimated heterogeneity at Level 2 and Level 3 that is explained by the measurement type; $\tau_{\left(Level\;2\right)}^{2}$, heterogeneity of effect sizes within studies; $\tau_{\left(Level\;3\right)}^{2}$, heterogeneity of effect sizes between studies*.

### Methodological Factors and Influential Studies

Where possible, we examined whether methodological factors of primary studies affected the relationships between rotation and the superordinate outcome categories (see Supplementary Material, Supplementary Tables 2–6, for detailed results). For all outcome categories, there were no significant differences between correlational and (quasi-)experimental studies, laboratory and field studies, and studies with a within- and between-subjects design. The comparison of cross-sectional with time-lagged studies showed significant differences for learning and development outcomes (*r*_Diff_ = 0.43, *p* = 0.01) and for physical health outcomes (*r*_Diff_ = −0.20, *p* = 0.05). The relationship between rotation and learning and development was stronger in cross-sectional studies, the relationship between rotation and physical health was stronger in time-lagged studies.

To determine whether single studies with very large sample sizes might have skewed the results of the meta-analysis, we conducted a sensitivity analysis. More specifically, we computed the relationships between rotation and the outcomes without the studies of Avgoustaki (2016; *n* = 29,537), Bouville and Alis (2014; *n* = 24,486), Kampkötter et al. (2016; *n*_1_ = 90,321; *n*_2_ = 91,987), and Ollo-Lopez et al. (2010; *n* = 12,056). The results showed that the exclusion of these studies affected the effect sizes only marginally (Supplementary Table 7).

## Discussion

Ambiguous results from previous studies required a quantitative integration to assess an average relationship between job and task rotation and the beneficial outcomes that organizations expect and textbooks assert. Based on theories and models from multiple disciplines, we had assumed that rotation was positively associated with various employee attitudes, learning and development outcomes, psychological health, and organizational performance. The results supported our assumptions regarding the positive relationships between rotation and job satisfaction, organizational commitment, career success, labor flexibility, general psychological health, individual performance, productivity, and less stress and burnout. We could, however, not find significant evidence for positive relationships between rotation and work motivation, job involvement, competence development, speed of product development, innovativeness, financial performance, and reduced turnover (intention).

Regarding the relationships between rotation and physical health outcomes, we had expected positive relationships between rotation and physical health when the reference group performed high-intensity work, and negative relationships when the reference group performed low-intensity work. The results indeed showed that rotation was associated with reduced musculoskeletal complaints and physical workload when compared to high-intensity work. When compared to low-intensity work, there were positive, yet non-significant, relationships with musculoskeletal complaints and physical workload. There were not enough studies to investigate the associations between rotation and general physical health.

A comparison of job and task rotation revealed that, as expected, task rotation resulted in stronger correlations with attitudes, whereas job rotation had stronger correlations with learning and development and organizational performance. Contrary to our expectations, job rotation was also more strongly correlated with psychological health outcomes. In each case, the difference between job rotation and task rotation was not significant, although the absolute values of the correlation coefficients differed greatly in most cases. For example, when compared to averaged effect sizes in the human resources and organizational behavior literatures (Paterson et al., 2016), the association between job rotation and learning and development exceeds in magnitude 50% of effect sizes, whereas the association between task rotation and learning and development exceeds only about 17%. There were not enough primary studies on relationships between job rotation and physical health so that we could not test our assumptions for this outcome category.

Lastly, as expected, we found that as the societal culture of the primary studies becomes more individualistic, the relationship between task rotation and employee attitudes decreases. We had also assumed the opposite for job rotation but could not find evidence for this assumption. We had thought that job rotation could be more beneficial for individual-oriented goals, such as career advancement, and therefore result in more favorable attitudes. However, it is possible that these individual-oriented goals are only relevant in the more distant future so that they do not affect more direct attitudinal responses.

The results of our exploratory research question showed that there were significant differences between subjectively and objectively measured outcomes. The association between rotation and physical health and organizational performance—the only outcome categories with enough objectively measured outcomes—was stronger when the outcomes were measured subjectively.

### Theoretical Implications

We aimed to explain the expected relationships between rotation and employee attitudes, learning and development, psychological and physical health, and organizational performance with the help of theories and models from the respective disciplines. Based on the results of the comparison of job rotation and task rotation, we could draw initial conclusions on the appropriateness of our theoretical arguments for the investigated outcomes.

Based on the job characteristics model (Hackman and Oldham, 1976), we had assumed that a positive association between rotation and employee attitudes could be explained by the fact that jobs with rotation usually provide certain work characteristics (e.g., task variety). These should be more prominent in task rotation than in job rotation because task rotation usually happens more frequently. Also, job rotation should be more likely to reduce the work characteristic of social support, as it usually involves a change to a different workplace. Hence, we assumed that if the association between attitudes and task rotation was stronger than between attitudes and job rotation, this would be a first indicator that the job characteristics model provided an appropriate explanation of the relationship between rotation and attitudes. The results supported this assumption.

In regard to learning and development, we drew on the work design growth model (Parker, 2017) and expected rotation to be beneficial because it broadened the employees' skills and perspectives. We believed that job rotation provided more diverse work environments than task rotation and thus more diverse perspectives that could stimulate learning and growth. The comparison of job and task rotation showed that there was indeed a stronger correlation between learning and job rotation. This finding can be regarded as a first confirmation that the work design growth model is an appropriate explanation for the relationship between rotation and learning and development.

Based on the integrative model of psychologically healthy workplaces of Kelloway and Day (2005), we had assumed that rotation improved psychological health because it reduces negative demands and stressors and promotes organizational resources. We had expected that task rotation would be more suitable than job rotation to provide resources, such as variety and opportunity for skill use, and reduce demands, such as an imbalanced workload. The results, however, indicated slightly stronger relationships between job rotation and psychological health. One explanation could be that in some cases, task rotation could be perceived as stressful because the workflow is interrupted. Fletcher et al. (2018) found, for instance, a positive relationship between workflow interruptions and psychological stress reactions.

Drawing on a model by Westgaard and Winkel (1996), we had expected a beneficial effect of rotation on physical health because rotation between activities that stress different body regions provides opportunities to recover. However, previous literature reviews (e.g., Leider et al., 2015; Padula et al., 2017) had found only weak or ambiguous relationships between rotation and physical health. Our results provide an explanation for these results: There is only a beneficial health effect of rotation when it is compared to high-intensity work. Thus, the model by Westgaard and Winkel (1996) is a fitting explanation for the relationship between rotation and physical health, as long as the rotation introduces more light-intensity work.

Lastly, based on resource-based theory (Barney, 1991; Barney et al., 2011), we expected rotation to be associated with organizational performance because it promotes workforce flexibility and organizational learning. Analogously to our expectations regarding learning and development, we believed that job rotation would yield stronger effects than task rotation. The results supported this assumption and therefore give a first indication that the resource-based theory provides a suitable explanation for the relationship between rotation and organizational performance.

### Practical Implications

The prevailing view of job and task rotation is that they provide a variety of advantages for organizations and employees. More than half of U.S. organizations practice job rotation (Cranet, 2017) and many textbooks recommend rotation as a work design technique. The results from this meta-analysis give reason to reconsider the unrestricted recommendation of rotation. First, although the relationships between rotation and its outcomes were positive on average, many correlations were non-significant and small. Thus, organizations planning to implement rotation should be aware that the intervention might not improve the targeted outcomes very much. On the basis of the existing primary studies, organizations can only expect great associations between rotation and job satisfaction, career success, labor flexibility, and general psychological health.

Second, depending on the desired outcome, organizations should also bear in mind that the concrete design of the rotation can potentially influence the relationship between rotation and its outcomes. More specifically, the results of our meta-analysis indicate that task rotation seems to be more suitable than job rotation when the desired outcomes are improved employee attitudes. Job rotation, however, should be preferred when the goal is an increase in employee learning and development, improved psychological health, or an increase in organizational performance. In addition, practitioners should carefully analyze future primary studies to determine whether they report on job or task rotation so that they can draw correct conclusions from these studies.

Third, with regard to physical health and organizational performance, organizations should be aware that subjectively measured outcomes were more strongly related to rotation. This is critical because the actual, objectively measurable benefit is highly relevant for these outcomes. It is probably a waste of resources to adopt a work design method that only improves the perceived innovativeness, individual performance, or physical workload. In comparison to that, the perceived stress or satisfaction in a workplace are measures where the subjective assessment might provide appropriate information.

### Limitations and Directions for Future Research

We believe that our meta-analysis provides important insights into the effects of job and task rotation. However, there are also some limitations. To begin with, the relatively small number of studies for some of the analyses prevents us from drawing wide generalizations. However, compared with alternative techniques of study aggregation (e.g., vote counting or narrative reviews), which are usually dependent on subjective and sometimes untransparent decisions, the meta-analytic integration of studies provides the advantage of a quantification of the average effect. Valentine et al. (2010) therefore came to the conclusion that a meta-analysis already provides added value when it is based on as few as two studies. Furthermore, by using a three-level meta-analysis, we included as much information as possible from each primary study.

A further limitation is that we could include only a relatively small number of unpublished primary studies. This is problematic because the results from the included studies might differ from the results of the overall research that has potentially been done on the effects of job and task rotation. The reason for a possible difference lies in publication bias, which describes the tendency that significant results and results that support the authors' hypotheses are more likely to be published (Rothstein et al., 2005). Thus, the averaged effect sizes reported in this meta-analysis might have been lower if we would have been able to include more unpublished data. On the other hand, a meta-meta-analysis of 83 meta-analyses published in *Psychological Bulletin* has found only weak evidence for publication bias and an overestimation of effect sizes in psychological meta-analyses (van Aert et al., 2019).

Additionally, most included primary studies had a cross-sectional, correlational design. This could be regarded as a limitation because these studies do not allow for conclusions to be drawn about causality. To find out more about the direction of the effect and to rule out alternative explanations, we recommend that further research with (quasi-)experimental designs be carried out.

Another limitation was that our moderator analyses were limited by the information provided in the primary studies. This meant that there were some moderators that we could not investigate. For instance, we were interested in whether the perceived similarity of tasks or jobs would moderate the relationships between rotation and beneficial outcomes. We believed that a greater similarity would weaken the relationships because it would result in less variety, provide less diverse stimuli from the work environment, could be perceived as more repetitive, and might not leave enough opportunity for muscle recovery.

Another group of moderators that might further explain heterogeneity can be derived from self-determination theory (Deci et al., 2017). The theory claims that every individual has basic human needs (i.e., the needs for autonomy, competence, and relatedness), which, when satisfied, result in internal motivation and consequently lead to psychological well-being and enhanced performance (Deci et al., 2017). The design of job and task rotation might satisfy these needs in some cases more than in others. One could, for instance, assume that having a say during the implementation of rotation strengthens its relationship with employee-related outcomes because this would satisfy the need for autonomy. In general, greater work autonomy is associated with more positive attitudes, greater job performance, and reduced stress and burnout (Humphrey et al., 2007). In order for job and task rotation to satisfy the need for competence, it might be necessary for the rotation to involve activities that require a diverse set of skills. Lastly, it might be possible that the rotation between workstations with varying colleagues is more beneficial than a rotation with limited potential for interaction because the latter alternative does not satisfy the need for relatedness. Studies that investigated job rotation between different functions in an organization found, for example, that the rotation was associated with more interdepartmental communication and cross-functional activities (Hauptman and Hirji, 1999).

### Conclusion

Job and task rotation have been a research topic in several disciplines for many years. This meta-analysis is the first to provide a quantitative estimate of the relationships between these work design methods and their expected outcomes, point to moderating factors, and clarify the differences between job rotation and task rotation. Our results showed that rotation was generally positively related to a variety of outcomes. However, many relationships were only small and non-significant. Positive relationships between rotation and physical health could only be found when rotation was compared to high-intensity work. A comparison of job and task rotation revealed that task rotation yielded stronger relationships with attitudinal outcomes, whereas job rotation had stronger relationships with development, psychological health, and organizational performance outcomes. Individualism led to weaker relationships between task rotation and attitudes, and relationships between rotation and physical health as well as organizational performance were stronger for subjective outcome measures. In conclusion, this meta-analysis enriches our understanding of job and task rotation because we showed that these two methods should not be confused, and that many expectations toward rotation cannot yet be empirically supported.

## Data Availability Statement

Publicly available datasets were analyzed in this study. This data can be found at: Open Science Framework, https://osf.io/xtrkn/.

## Author Contributions

LM and GM contributed to the conception and design of the meta-analysis, contributed to the manuscript revision, read, and approved the submitted version. LM conducted the literature search, coding of studies, performed the statistical analyses, and wrote the manuscript.

## Conflict of Interest

The authors declare that the research was conducted in the absence of any commercial or financial relationships that could be construed as a potential conflict of interest.

## Footnotes

^1^https://osf.io/xtrkn/

^2^https://www.siop.org/Career-Center/Calls-and-Announcements

^3^https://www.linkedin.com/groups/1999015/

^4^https://translate.google.com
