# Supplementary material for: More Hype Than Substance? A Meta-Analysis on Job and Task Rotation
Source: Front Psychol. 2021 Mar 25;12:633530. doi: 10.3389/fpsyg.2021.633530 (PMC8044787; doi:10.3389/fpsyg.2021.633530)
Supplement: Supplementary file 1 [file Data_Sheet_1.pdf]

Supplementary Material

1 Synthetic Outcome Constructs and Corresponding Operationalizations

Supplementary Table 1. Synthetic Outcome Constructs and Corresponding Operationalizations

(Synthetic) construct	Operationalization
Job satisfaction	Job satisfaction
Work motivation	Work motivation
Job involvement	Job/task involvement
	Resignation (recoded)
	Extensive work effort
	Voluntary effort
Organizational commitment	Organizational commitment
	Affective duty commitment
Competence development	Satisfaction of need for competence
	Problem-solving demand
	Skill improvement
Career success	Subjective career success
	Career management and talent management effectiveness
	Promotion rate
	Salary growth
Labor flexibility	Labor flexibility acquired/implemented
	Adaptability
	Job specialization (recoded)
General psychological health	General psychological health
	Positive affect
	Negative affect (recoded)

(Synthetic) construct	Operationalization
Stress and burnout	Stress
	Emotional and cognitive strain
	Depression
	Mental exhaustion
	Burnout
	Work engagement (recoded)
	Lack of energy
	Fatigue
	Need for recovery
General physical health	General health
	Work ability
	Take days off (recoded)
	Cases of illness (recoded)
Musculoskeletal complaints	Musculoskeletal complaints
	Musculoskeletal disorders (e.g., carpal tunnel syndrome)
	Rating of perceived discomfort in extremities
	Pain
Physical workload	Physical exhaustion
	Heart rate
	Rating of perceived (physical) fatigue
Individual performance	Subjective performance
	Individual productivity
Productivity	Productivity
	Utilization rate
Speed of product development	Speed of new product development
	Flexibility
Innovativeness	Product innovation
	Process innovation
	Quality innovation

(Synthetic) construct	Operationalization
Financial performance	Cost reduction
	Profitability
	Sales growth
	Organizational crisis (recoded)
Turnover (intention)	Turnover
	Turnover intention
	Intent to stay (recoded)

2 Influence of Methodological Factors

Analyses of methodological factors were conducted when the subsamples contained at least three studies.

Supplementary Table 2. Relationships Between Job and Task Rotation and Attitudes Moderated by Different Methodological Factors

Chronological order									
					CI 95%				
Chronological order	k	n_{es}	Estimate	SE	LL	UL	Z	p	R^2
Time-lagged	3	7	.17	.12	–.07	.40	1.40	.16	
Cross-sectional	15	34	.12	.05	.02	.22	2.39	.02	
$\tau^2_{(Level\ 2)}$			.01	.01	.00	.02	2.34	.02	.00
$\tau^2_{(Level\ 3)}$			.03	.01	–.00	.06	1.84	.07	.03
Study rigor									
					CI 95%				
Study rigor	k	n_{es}	Estimate	SE	LL	UL	Z	p	R^2
(Quasi-)experimental	3	7	.17	.12	–.07	.40	1.40	.16	
Correlational	15	34	.12	.05	.02	.22	2.39	.02	
$\tau^2_{(Level\ 2)}$			.01	.01	.00	.02	2.34	.02	.00
$\tau^2_{(Level\ 3)}$			.03	.01	–.00	.06	1.84	.07	.03
Study setting									
					CI 95%				
Study setting	k	n_{es}	Estimate	SE	LL	UL	Z	p	R^2
Laboratory	3	7	.17	.12	–.07	.40	1.40	.16	
Field	15	34	.12	.05	.02	.22	2.39	.02	
$\tau^2_{(Level\ 2)}$			.01	.01	.00	.02	2.34	.02	.00
$\tau^2_{(Level\ 3)}$			.03	.01	–.00	.06	1.84	.07	.03

Note. k = number of independent samples; n_{es} = number of effect sizes; CI = confidence interval; LL = lower level; UL = upper level; Z = Wald approximation; R^2 = estimated heterogeneity at Level 2 and Level 3 that is explained by the methodological factors; $\tau^2_{(Level\ 2)}$ = heterogeneity of effect sizes within studies; $\tau^2_{(Level\ 3)}$ = heterogeneity of effect sizes between studies.

Supplementary Table 3. Relationships Between Job and Task Rotation and Learning and Development Moderated by Different Methodological Factors

Chronological order									
					CI 95%				
Chronological order	k	n_{es}	Estimate	SE	LL	UL	Z	p	R^2
Time-lagged	3	3	–.09	.14	–.36	.17	–0.68	.50	
Cross-sectional	6	13	.33	.09	.16	.51	3.79	< .001	
$\tau^2_{(Level\ 2)}$			.01	.01	–.00	.02	1.26	.21	.00
$\tau^2_{(Level\ 3)}$			.04	.02	–.01	.09	1.59	.11	.49

Note. k = number of independent samples; n_{es} = number of effect sizes; CI = confidence interval; LL = lower level; UL = upper level; Z = Wald approximation; R^2 = estimated heterogeneity at Level 2 and Level 3 that is explained by the methodological factors; $\tau^2_{(Level\ 2)}$ = heterogeneity of effect sizes within studies; $\tau^2_{(Level\ 3)}$ = heterogeneity of effect sizes between studies.

Supplementary Table 4. Relationships Between Job and Task Rotation and Psychological Health Moderated by Different Methodological Factors

Chronological order									
					CI 95%				
Chronological order	k	n_{es}	Estimate	SE	LL	UL	Z	p	R^2
Time-lagged	6	10	.13	.06	.09	.26	2.11	.03	
Cross-sectional	7	14	.14	.05	.05	.24	2.88	.004	
$\tau^2_{(Level\ 2)}$			.00	–	–	–	–	–	.00
$\tau^2_{(Level\ 3)}$			.02	.01	.00	.03	2.15	.03	.00
Study rigor									
					CI 95%				
Study rigor	k	n_{es}	Estimate	SE	LL	UL	Z	p	R^2
(Quasi-)experimental	6	10	.13	.06	.01	.26	2.11	.03	
Correlational	7	14	.14	.05	.05	.24	2.88	.004	
$\tau^2_{(Level\ 2)}$			.00	–	–	–	–	–	.00
$\tau^2_{(Level\ 3)}$			.02	.01	.00	.03	2.15	.03	.00
Study setting									
					CI 95%				
Study setting	k	n_{es}	Estimate	SE	LL	UL	Z	p	R^2
Laboratory	3	5	.14	.09	–.04	.31	1.50	.13	
Field	10	19	.14	.04	.05	.13	3.23	.001	
$\tau^2_{(Level\ 2)}$			.00	–	–	–	–	–	.00
$\tau^2_{(Level\ 3)}$			.02	.01	.00	.03	2.15	.03	.00

Note. k = number of independent samples; n_{es} = number of effect sizes; CI = confidence interval; LL = lower level; UL = upper level; Z = Wald approximation; R^2 = estimated heterogeneity at Level 2 and Level 3 that is explained by the methodological factors; $\tau^2_{(Level\ 2)}$ = heterogeneity of effect sizes within studies; $\tau^2_{(Level\ 3)}$ = heterogeneity of effect sizes between studies.

Supplementary Table 5. Relationships Between Job and Task Rotation and Physical Health Moderated by Different Methodological Factors

Chronological order									
					CI 95%				
Chronological order	*k*	n_{es}	Estimate	*SE*	LL	UL	*Z*	*p*	R^2
Time-lagged	8	35	.26	.07	.11	.40	3.49	< .001	
Cross-sectional	8	56	.05	.07	–.09	.19	0.76	.45	
$\tau^2_{(Level\ 2)}$			.02	.00	.01	.03	5.02	< .001	.00
$\tau^2_{(Level\ 3)}$			.03	.01	.00	.05	2.17	.03	.28
**Study rigor**									
					CI 95%				
Study rigor	*k*	n_{es}	Estimate	*SE*	LL	UL	*Z*	*p*	R^2
(Quasi-)experimental	7	34	.26	.08	.11	.41	3.36	< .001	
Correlational	9	57	.07	.07	–.07	.20	0.95	.34	
$\tau^2_{(Level\ 2)}$			.02	.00	.01	.03	5.02	< .001	.00
$\tau^2_{(Level\ 3)}$			.03	.01	.00	.05	2.17	.03	.27
**Study setting**									
					CI 95%				
Study setting	*k*	n_{es}	Estimate	*SE*	LL	UL	*Z*	*p*	R^2
Laboratory	4	19	.19	.11	–.03	.40	1.71	.09	
Field	12	72	.14	.07	.01	.27	2.04	.04	
$\tau^2_{(Level\ 2)}$			.02	.00	.01	.03	5.03	< .001	.00
$\tau^2_{(Level\ 3)}$			.04	.02	.01	.07	2.40	.01	.01
**Study design**									
					CI 95%				
Study design	*k*	n_{es}	Estimate	*SE*	LL	UL	*Z*	*p*	R^2
Within-subjects	5	36	.07	.10	–.13	.27	0.70	.49	
Between-subjects	11	71	.14	.07	.01	.28	2.08	.04	
$\tau^2_{(Level\ 2)}$			.03	.01	.02	.04	5.13	< .001	.00
$\tau^2_{(Level\ 3)}$			.04	.02	.01	.07	2.32	.02	.03

Note. k = number of independent samples; n_{es} = number of effect sizes; CI = confidence interval; LL = lower level; UL = upper level; Z = Wald approximation; R^2 = estimated heterogeneity at Level 2 and Level 3 that is explained by the methodological factors; $\tau^2_{(Level\ 2)}$ = heterogeneity of effect sizes within studies; $\tau^2_{(Level\ 3)}$ = heterogeneity of effect sizes between studies. Effect sizes that compared rotation to low-intensity work were excluded in the analyses.

Supplementary Table 6. Relationships Between Job and Task Rotation and Organizational Performance Moderated by Different Methodological Factors

Chronological order									
					CI 95%				
Chronological order	*k*	n_{es}	Estimate	*SE*	LL	UL	*Z*	*p*	R^2
Time-lagged	9	18	.05	.05	–.05	.14	0.94	.35	
Cross-sectional	21	47	.13	.04	.05	.21	3.27	.001	
$\tau^2_{(Level\ 2)}$			.03	.01	.01	.04	4.06	< .001	.06
$\tau^2_{(Level\ 3)}$			.00	.00	–.01	.01	0.80	.42	.00
Study rigor									
					CI 95%				
Study rigor	*k*	n_{es}	Estimate	*SE*	LL	UL	*Z*	*p*	R^2
(Quasi-)experimental	5	11	.08	.07	–.06	.22	1.17	.24	
Correlational	25	45	.10	.04	.03	.17	2.80	.01	
$\tau^2_{(Level\ 2)}$			.03	.01	.01	.04	4.03	< .001	.00
$\tau^2_{(Level\ 3)}$			.00	.01	–.01	.01	0.52	.60	.00
Study setting									
					CI 95%				
Study setting	*k*	n_{es}	Estimate	*SE*	LL	UL	*Z*	*p*	R^2
Laboratory	4	10	.07	.08	–.08	.22	0.96	.34	
Field	26	55	.10	.04	.03	.17	2.89	.004	
$\tau^2_{(Level\ 2)}$			.03	.01	.01	.01	4.05	< .001	.00
$\tau^2_{(Level\ 3)}$			.00	.01	–.01	.01	0.54	.59	.00

Note. k = number of independent samples; n_{es} = number of effect sizes; CI = confidence interval; LL = lower level; UL = upper level; Z = Wald approximation; R^2 = estimated heterogeneity at Level 2 and Level 3 that is explained by the methodological factors; $\tau^2_{(Level\ 2)}$ = heterogeneity of effect sizes within studies; $\tau^2_{(Level\ 3)}$ = heterogeneity of effect sizes between studies.

Supplementary Table 7. Results of Sensitivity Analysis

Outcomes	*r*	r_s
Attitudes		
Job satisfaction	.27	.30
Work motivation	.12	.13
Job involvement	.10	.13
Organizational commitment	.16	.17
Learning & development		
Competence development	.13	.14
Career success	.31	.32
Labor flexibility	.32	.33
Psychological health		
General psychological health	.20	.21
Stress and burnout[a]	.13	.14
Physical health		
General physical health	.12	.13
Musculoskeletal complaints[a]	.08	.10
Physical workload[a]	.13	.14
Organizational performance		
Individual performance	.13	.16
Productivity	.13	.15
Speed of product development	.17	.17
Innovativeness	.12	.12
Financial performance	.13	.13
Turnover (intention)[a]	.12	.13

Note. *r* = effect size estimate of meta-analysis with all studies; r_s = effect size estimate of meta-analysis without studies with extremely large effect sizes.

[a]Reverse-coded; high values indicate low levels of stress and burnout, musculoskeletal complaints, physical workload, and turnover (intention)

Supplementary Table 8. PRISMA Checklist

Section/topic	#	Checklist item	Reported on page #
**TITLE**			
Title	1	Identify the report as a systematic review, meta-analysis, or both.	1
**ABSTRACT**			
Structured summary	2	Provide a structured summary including, as applicable: background; objectives; data sources; study eligibility criteria, participants, and interventions; study appraisal and synthesis methods; results; limitations; conclusions and implications of key findings; systematic review registration number.	1
**INTRODUCTION**			
Rationale	3	Describe the rationale for the review in the context of what is already known.	2
Objectives	4	Provide an explicit statement of questions being addressed with reference to participants, interventions, comparisons, outcomes, and study design (PICOS).	2-3
**METHODS**			
Protocol and registration	5	Indicate if a review protocol exists, if and where it can be accessed (e.g., Web address), and, if available, provide registration information including registration number.	N/A
Eligibility criteria	6	Specify study characteristics (e.g., PICOS, length of follow-up) and report characteristics (e.g., years considered, language, publication status) used as criteria for eligibility, giving rationale.	9-10
Information sources	7	Describe all information sources (e.g., databases with dates of coverage, contact with study authors to identify additional studies) in the search and date last searched.	9-10
Search	8	Present full electronic search strategy for at least one database, including any limits used, such that it could be repeated.	9
Study selection	9	State the process for selecting studies (i.e., screening, eligibility, included in systematic review, and, if applicable, included in the meta-analysis).	10 + flow chart
Data collection process	10	Describe method of data extraction from reports (e.g., piloted forms, independently, in duplicate) and any processes for obtaining and confirming data from investigators.	10

Section/topic	#	Checklist item	Reported on page #
Data items	11	List and define all variables for which data were sought (e.g., PICOS, funding sources) and any assumptions and simplifications made.	11 + suppl. material
Risk of bias in individual studies	12	Describe methods used for assessing risk of bias of individual studies (including specification of whether this was done at the study or outcome level), and how this information is to be used in any data synthesis.	12
Summary measures	13	State the principal summary measures (e.g., risk ratio, difference in means).	10
Synthesis of results	14	Describe the methods of handling data and combining results of studies, if done, including measures of consistency (e.g., I^2) for each meta-analysis.	11-12
Risk of bias across studies	15	Specify any assessment of risk of bias that may affect the cumulative evidence (e.g., publication bias, selective reporting within studies).	14
Additional analyses	16	Describe methods of additional analyses (e.g., sensitivity or subgroup analyses, meta-regression), if done, indicating which were pre-specified.	14
**RESULTS**			
Study selection	17	Give numbers of studies screened, assessed for eligibility, and included in the review, with reasons for exclusions at each stage, ideally with a flow diagram.	Flow chart
Study characteristics	18	For each study, present characteristics for which data were extracted (e.g., study size, PICOS, follow-up period) and provide the citations.	Suppl. Table 9
Risk of bias within studies	19	Present data on risk of bias of each study and, if available, any outcome level assessment (see item 12).	Suppl. Table 9
Results of individual studies	20	For all outcomes considered (benefits or harms), present, for each study: (a) simple summary data for each intervention group (b) effect estimates and confidence intervals, ideally with a forest plot.	Suppl. Table 9
Synthesis of results	21	Present results of each meta-analysis done, including confidence intervals and measures of consistency.	28-31
Risk of bias across studies	22	Present results of any assessment of risk of bias across studies (see Item 15).	14 + suppl. material

Section/topic	#	Checklist item	Reported on page #
Additional analysis	23	Give results of additional analyses, if done (e.g., sensitivity or subgroup analyses, meta-regression [see Item 16]).	14
**DISCUSSION**			
Summary of evidence	24	Summarize the main findings including the strength of evidence for each main outcome; consider their relevance to key groups (e.g., healthcare providers, users, and policy makers).	14-15
Limitations	25	Discuss limitations at study and outcome level (e.g., risk of bias), and at review-level (e.g., incomplete retrieval of identified research, reporting bias).	17-18
Conclusions	26	Provide a general interpretation of the results in the context of other evidence, and implications for future research.	16-18
**FUNDING**			
Funding	27	Describe sources of funding for the systematic review and other support (e.g., supply of data); role of funders for the systematic review.	19
