# Supplementary material for: More Hype Than Substance? A Meta-Analysis on Job and Task Rotation
Source: Front Psychol. 2021 Mar 25;12:633530. doi: 10.3389/fpsyg.2021.633530 (PMC8044787; doi:10.3389/fpsyg.2021.633530)
Supplement: Supplementary file 3 [file Table_9.pdf]

Supplementary Table 9. Overview of Studies Included in the Meta-Analysis

Authors	Year	*N*	Study rigor	Intervention	Operationalization	Outcome (original wording)	Operationalization	*r*
Arasi & Aghdam	2016	330	correlational	NA	Questionnaire developed by previous study (no examples given)	Organizational commitment (affective duty commitment)	Questionnaire developed by Mowday et al. (1979) and Meyer and Allen (1997)	.33
Avgoustaki	2016	29,537	correlational	Task rotation	Does your job involve rotating tasks between yourself and colleagues? (yes/no)	Job involvement (extensive work effort)	How many times a month do you work more than 10 hours a day?	.06
Balogh et al.	2016	828	correlational	Task rotation	Observation of work tasks: cashier work, picking work, delicatessen work, and mixed work	Musculoskeletal complaints (neck/shoulder complaints)	Standardized Nordic Questionnaire (Kuorinka et al., 1987)	–.09
						Musculoskeletal complaints (elbow/hand complaints)	Standardized Nordic Questionnaire (Kuorinka et al., 1987)	.02
Bao et al.	2016	1,834	correlational	Task rotation	Interview and observation: dichotomized variable whether worker was assigned with more than one single task for a period of time in their job during a work shift	Job satisfaction	All in all, how satisfied are you with your job?	–.13
						Organizational commitment (recommend job to others)	How strongly would you recommend your job to someone else?	–.07
						Organizational commitment (take this job again)	If you were looking for a new job now, how likely is it that you would decide to take this job again?	–.13
						Physical workload (physical exhaustion)	How often are you physically exhausted after work?	–.00
						General physical health (general health)	How would you describe your general health compared to others of your own age?	–.01
						Stress and burnout (depressed)	How often during the past year have you felt "down", blue, or depressed?	–.04
						Stress and burnout (mental exhaustion)	How often are you mentally echausted after work?	–.03
Bodin	2011	3,710	correlational	Both	Job/task rotation ≥ once per week (yes/no)	Musculoskeletal complaints (shoulder pain with or without rotator cuff syndrome)	Physical examination	.04

Authors	Year	*N*	Study rigor	Intervention	Operationalization	Outcome (original wording)	Operationalization	*r*
Bouville & Alis	2014	24,486	correlational	Task rotation	Do you occupy different workstations? (yes/no)	Job satisfaction	On the whole, I am satisfied with my job.	–.06
						Turnover intention (intent to stay)	Do you intend to change work positions or jobs?	.08
						Competence development (problem-solving demand)	In your work, have you had the possibility to solve a problem, should one arise? (yes/no)	–.04
						General physical health	Self-developed items (e.g., my work is rather harmful to my health)	–.06
Campion et al.	1994	255	correlational	Job rotation	Number of rotations (defined as change in job title or department without increase in salary) divided by number of years employed in the organization	Individual performance	Personnel records	.25
						Career success (promotion rate)	Number of promotions divided by number of years of tenure	.37
						Career success (salary growth)	Current salary class minus salary class at entry into the company divided by number of years of tenure	.29
						Career success (career affect)	Extent of agreement that career affect was benefit of job rotation	.17
						Competence development (administrative skills improvement)	Judgment of the extent to which job rotation generally improved each knowledge and skill	.24
						Competence development (technical skills improvement)	Judgment of the extent to which job rotation generally improved each knowledge and skill	.01
						Competence development (business skills improvement)	Judgment of the extent to which job rotation generally improved each knowledge and skill	.27
						Competence development (personal development)	Extent of agreement that personal development was benefit of job rotation	.20
Choe	2004	93	correlational	Job rotation	Questionnaire developed by previous studies (e.g., rotating jobs among production employees and between production and other departments)	Financial performance (cost reduction)	Self-developed items	.22
						Speed of product development (flexibility)	Self-developed items	.24
Colombo et al.	2007	763	correlational	Both	Do operators rotate across jobs or tasks on the line? (yes/no)	Financial performance (profitability)	Company data on returns on investment	.01

Authors	Year	*N*	Study rigor	Intervention	Operationalization	Outcome (original wording)	Operationalization	*r*
Comper et al.	2017	368	(quasi-)experimental	Task rotation	Intervention: participants rotated between tasks (experimental condition) or not (control condition)	General physical health	WHO Quality of Life Scale (Fleck et al. 2000)	–.01
						Musculoskeletal complaints (working hours lost due to sick leave resulting from musculoskeletal diseases)	Sick notes	–.09
						General psychological health	WHO Quality of Life Scale (Fleck et al. 2000)	.04
						Stress and burnout (fatigue)	Need for Recovery Scale (Moriguchi et al. 2010)	–.03
						Individual performance (productivity)	WHO Health and Work Performance Questionnaire (Kessler et al., 2003)	.14
Cruz & Pil	2011	1,708	correlational	Both	Managers indicated whether team members rotated tasks or roles (yes/no)	Stress and burnout	Warr's (1990) measure of job anxiety-contentment	–.07
de Clerq et al.	2013	232	correlational	Job rotation	Questionnaire developed by previous studies (e.g., extent to which employees rotate across functional areas)	Innovativeness (product innovativeness)	Items adapted from previous study (e.g., extent to which employees rotate across functional areas)	.16
de Leeuw & Wiers	2015	52	correlational	NA	Questionnaire about the application of manpower planning strategies	Productivity (utilization)	NA	.42
						Productivity	NA	.31
Delobbe & Vandenberghe	2001	201	correlational	Job rotation	Objective data: number of different jobs during the onboarding of new employees	Organizational commitment	Questionnaire developed by Allen & Meyer (1990)	.15
						Job satisfaction	Items adapted from previous study (e.g., overall, I am satisfied with my job)	.22
						Turnover intention	Items adapted from previous study (e.g., I often think about leaving this organization)	–.12
Dinis & Fronteira	2015	55	correlational	Job rotation	Number of work shifts carried out in different units	Job satisfaction	Questionnaire developed by Graça (1999)	–.43 – .18[a]

Authors	Year	*N*	Study rigor	Intervention	Operationalization	Outcome (original wording)	Operationalization	*r*
Godard	2001	508	correlational	NA	Is there a job rotation program? (yes/no)	Job involvement (task involvement)	Items adapted from previous studies (e.g., the work you do is meaningful to you)	.03
						Stress and burnout (stressfulness)	Items adapted from previous studies (e.g., your job is stressful)	.01
						Stress and burnout (fatigue)	Self-developed items (e.g., you are often worn out by the end of the day)	–.02
						Job satisfaction	Michigan Organizational Assessment Package (Institute for Social Research 1975)	.02
						Organizational commitment	Organizational Commitment Questionnaire (Mowday et al., 1982)	.04
						Work motivation	Self-developed items (e.g., you are highly committed to do the best job you can)	–.01
Han et al.	2020	985	correlational	Job rotation	Whether or not employees did job rotation in low altitude	General physical health (hyperhemoglobin)	Physical examination	.17
He et al.	2016	725	correlational	Job rotation	Items adapted from established scale (e.g., co-location of design engineers and manufacturing managers)	Speed of new product development	Items from previous studies (no examples given)	.19

Authors	Year	N	Study rigor	Intervention	Operationalization	Outcome (original wording)	Operationalization	r
Horton et al.	2012	12	(quasi-)experimental	Task rotation	Experiment in which participants rotated between tasks with low and high exertion levels (experimental condition) or performed only one task (control condition)	Physical workload (muscle fatigue)	Objective physical data: electromyographic amplitude	.76 / –.59[b]
						Physical workload (muscle fatigue)	Objective physical data: electromyographic mean power frequency	–.17 / .28[b]
						Physical workload (muscle fatigue)	Objective physical data: Dimitrov Spectral Index	.26 / –.27[b]
						Musculoskeletal complaints (rating of perceived discomfort shoulder)	Borg scale (Borg, 1990)	.24 / –.28[b]
						Musculoskeletal complaints (rating of perceived discomfort upper arm)	Borg scale (Borg, 1990)	.16 / –.32[b]
						Musculoskeletal complaints (rating of perceived discomfort upper back)	Borg scale (Borg, 1990)	.16 / –.32[b]
						Individual performance (moment fluctuations)	Errors during task execution	.00 / .11[b]
						Individual performance (sample entropy)	Errors during task execution	–.64 / .44[b]
						Individual performance (peak error)	Errors during task execution	–.04 / .11[b]
Horton et al.	2013	12	(quasi-)experimental	Task rotation	Experiment in which participants rotated between tasks with low and high exertion levels (experimental condition) or performed only one task (control condition)	Musculoskeletal complaints (rating of perceived discomfort shoulder)	Borg scale (Borg, 1990)	.26 / –.51[b]
						Musculoskeletal complaints (rating of perceived discomfort upper arm)	Borg scale (Borg, 1990)	.12 / –.21[b]
						Musculoskeletal complaints (rating of perceived discomfort upper back)	Borg scale (Borg, 1990)	.03 / –.31[b]
						Physical workload (muscle fatigue)	Objective physical data: electromyographic mean power frequency	–.22 / .11[b]
						Physical workload (heart rate)	Percentage of heart rate reserve	.12 / .00[b]
						Individual performance	Completed number of assemblies	–.16 / .09[b]

Authors	Year	N	Study rigor	Intervention	Operationalization	Outcome (original wording)	Operationalization	r
Horton et al.	2015	12	(quasi-)experimental	Task rotation	Experiment in which participants rotated between tasks with low and high exertion levels (experimental condition) or performed only one task (control condition)	Musculoskeletal complaints (rating of perceived discomfort lower back)	Borg scale (Borg, 1990)	.19 / –.22[b]
						Musculoskeletal complaints (rating of perceived discomfort upper back)	Borg scale (Borg, 1990)	.08 / –.15[b]
						Musculoskeletal complaints (rating of perceived discomfort shoulder)	Borg scale (Borg, 1990)	.17 / –.19[b]
						Musculoskeletal complaints (rating of perceived discomfort upper arm)	Borg scale (Borg, 1990)	.23 / –.21[b]
						Physical workload (heart rate)	Percentage of heart rate reserve	.33 / –.38[b]
Hsieh & Chao	2004	304	correlational	Job rotation	Items adapted from established scale (no examples given)	Stress and burnout (exhaustion)	Maslach Burnout Inventory (Maslach et al., 1996)	–.11
						Stress and burnout (cynicism)	Maslach Burnout Inventory (Maslach et al., 1996)	–.02
						Stress and burnout (professional efficacy)	Maslach Burnout Inventory (Maslach et al., 1996)	.09
						Labor flexibility (job specialization)	Self-developed items based on previous studies (e.g., what do you think of your job techniques or skills)	.33
Iwakiri et al.	2016	347	correlational	NA	Work rotation (doing/not doing)	Musculoskeletal complaints (low back pain)	Self-report of low back pain	–.20
Jeon & Jeong	2013	1,115	correlational	Task rotation	Self-classification into one of the clusters hourly, daily, weekly, monthly rotation between 8, 20, or 23 tasks (or no rotation)	Musculoskeletal complaints (musculoskeletal patient rate)	Self-developed item (not indicated)	.02 – .41[a]
						Musculoskeletal complaints (sick leave due to musculoskeletal disorders)	Self-developed item (not indicated)	–.06 – .53[a]

Authors	Year	N	Study rigor	Intervention	Operationalization	Outcome (original wording)	Operationalization	r
Jeon & Jeong	2016	1,115	correlational	Task rotation	Self-classification into one of the clusters hourly, daily, weekly, monthly rotation between 8, 20, or 23 tasks (or no rotation)	Job satisfaction	Self-developed item (not indicated)	.02 – .51[a]
						Musculoskeletal complaints (musculoskeletal disorder rate)	Self-developed item (not indicated)	–.21 – .14[a]
						Musculoskeletal complaints (subjective musculoskeletal disorder prevention)	Self-developed item (not indicated)	–.34 – .18[a]
						Productivity	Self-developed item (not indicated)	–.38 – .13[a]
						Productivity (subjective)	Self-developed item (not indicated)	–.14 – .32[a]
Jones & James	2018	16	(quasi-)experimental	Task rotation	Intervention, in which experimental group rotated between five main tasks in an underground coal mine (e.g., right bolter, supplies)	General physical health	WHO Quality of Life scale Australian edition (Murphy et al., 2000)	.03
						General psychological health	WHO Quality of Life Scale Australian edition (Murphy et al., 2000)	.43
						Stress and burnout (fatigue)	Need for Recovery after Work Scale (Sluiter et al., 2003)	–.32
Kampkötter et al.	2016	90,321	correlational	Job rotation	Rotation as given when employee worked in a different function in year 2005 than in year 2004	Individual performance (performance rank)	Rank position of the respective employee in the relative bonus distribution in their work unit	.01
						Individual performance (bonus payment)	Company data of annual, short-term performance-related bonus payment	.02
		91,987	correlational	Job rotation	Rotation as given when employee worked in a different function in year 2005 than in year 2004	Individual performance (performance rank)	Rank position of the respective employee in the relative bonus distribution in their work unit	.00
						Individual performance (bonus payment)	Company data of annual, short-term performance-related bonus payment	–.02
Khan et al.	2016	435	correlational	NA	NA	Organizational commitment	Allen and Meyer (1990)	.70
						Individual performance	Self-developed items (no example given)	.69

Authors	Year	N	Study rigor	Intervention	Operationalization	Outcome (original wording)	Operationalization	r
Kim et al.	2016	246	correlational	Job rotation	Self-developed questionnaire (e.g., interdepartmental transfer within the organization)	Stress and burnout (job engagement – vigor)	Questionnaire developed by Schaufeli et al. (2002)	–.21
						Stress and burnout (job engagement – dedication)	Questionnaire developed by Schaufeli et al. (2002)	–.25
						Stress and burnout (burnout – exhaustion)	Questionnaire developed by Schaufeli et al. (2002)	–.29
						Stress and burnout (burnout – cynicism)	Questionnaire developed by Schaufeli et al. (2002)	–.28
						Turnover intention	Items based on previous studies (e.g., I have considered changing my job seriously before)	–.24
Koncar et al.	2020	325	correlational	Job rotation	Degree of openness, possibilities and management support for changing a workplace within the company	General psychological health (welfare)	Self-developed item (no example given)	.43
Kuijer et al.	1999	8	(quasi-)experimental	Task rotation	Intervention: experimental group rotated between two of three tasks (e.g., refuse collecting and street sweeping), control group performed only one task	Physical workload (rating of perceived fatigue)	Questionnaire developed by Borg and Borg (1987)	–.80 – (–.21)[a]
						Physical workload (rating of perveiced exertion)	Questionnaire developed by Meikman et al. (1986)	–.85 – (–.51)[a]
						Physical workload (heart rate reserve)	Objective data of percentage of heart rate reserve	–.90 – .12[a]
Kuijer et al.	2005	67	(quasi-)experimental	Task rotation	Intervention: experimental group rotated between two tasks (refuse collecting and truck driving), control group performed only one task	Stress and burnout (need for recovery)	Questionnaire developed by Van Veldhoven and Meijman (1994)	–.02
Le Meunier-Fitzhugh & Massey	2019	146	correlational	Job rotation	There is an opportunity for sales and marketing staff to transfer between departments (not at all – an extreme extent)	Financial performance (business performance)	Items adapted from previous study (e.g., how successful is the organization at generating a high level of sales revenue?)	.25
Leenders & Wierenga	2002	148	correlational	Job rotation	Proportion of managers involved in cross-functional job rotation	Speed of new product development (new product performance)	Items based on previous studies (e.g., the speed at which new products are developed)	.11

Authors	Year	*N*	Study rigor	Intervention	Operationalization	Outcome (original wording)	Operationalization	*r*
Luger et al.	2016	10	(quasi-)experimental	Task rotation	Experiment in which participants rotated between static and dynamic tasks (experimental condition) or performed only one task (control condition)	Stress and burnout (lack of energy)	SOFI Swedish Occupational Fatigue Inventory (Ahsberg et al., 1997)	–.09
						Physical workload (physical exertion)	SOFI Swedish Occupational Fatigue Inventory (Ahsberg et al., 1997)	–.20
						Physical workload (sleepiness)	SOFI Swedish Occupational Fatigue Inventory (Ahsberg et al., 1997)	.10
						Musculoskeletal complaints (physical discomfort)	SOFI Swedish Occupational Fatigue Inventory (Ahsberg et al., 1997)	.00
						Work motivation (lack of motivation)	SOFI Swedish Occupational Fatigue Inventory (Ahsberg et al., 1997)	–.02
Martini & Cavenago	2017	558	correlational	Task rotation	I performed different tasks in different departments within the same company (no scale indicated)	Career success	Items based on previous studies (e.g., I am satisfied with the progress I have made toward meeting my goals for the development of new knowledge and skills)	.30
Mlekus et al.	2018	135	(quasi-)experimental	Task rotation	Vignette that described a workplace at which worker either rotated between four different tasks (experimental condition) or performed only one task (control condition)	Competence development (satisfaction of need for competence)	Work-Related Basic Need Satisfaction Scale (van den Broeck et al., 2010)	.13
						Job satisfaction	Items developed by Neuberger and Allerbeck (1978)	.25
						Job satisfaction	Items developed by Haarhaus (2016)	.25
						General psychological health (positive affect, negative affect)	Positive and Negative Affect Schedule (Watson et al., 1988)	.20
						Work motivation	Multidimensional Work Motivation Scale (Gagné et al., 2015)	.21
						Individual performance	Self-developed item (on a scale from 1 to 10, how high do you estimate your performance would be in the described job as a production mechanic based on your maximum performance capacity?)	.16

Authors	Year	*N*	Study rigor	Intervention	Operationalization	Outcome (original wording)	Operationalization	*r*
		159	(quasi-)experimental	Task rotation	Vignette that described a workplace at which worker either rotated between four different tasks (experimental condition) or performed only one task (control condition)	Competence development (satisfaction of need for competence)	Work-Related Basic Need Satisfaction Scale (van den Broeck et al., 2010)	–.04
						Job satisfaction	Items developed by Neuberger and Allerbeck (1978)	.18
						Job satisfaction	Items developed by and Haarhaus (2016)	.20
						General psychological health (positive affect, negative affect)	Positive and Negative Affect Schedule (Watson et al., 1988)	.11
						Work motivation	Multidimensional Work Motivation Scale (Gagné et al., 2015)	.13
						Individual performance	Self-developed item (on a scale from 1 to 10, how high do you estimate your performance would be in the described job as a production mechanic based on your maximum performance capacity?)	.17
Mohsan et al.	2012	285	correlational	NA	NA	Work motivation	Items based on previous studies	–.17
						Organizational commitment	Items adopted from Mowday and Steers (1979)	.23
						Job involvement	Items adopted from Uygur and Kilic (2009)	.16
Muramatsu et al.	1982	88	correlational	NA	NA	Work motivation	Self-developed items (no example given)	.44
Nasiripour et al.	2009	90	correlational	NA	NA	Individual performance	NA	.01
Ngirande & Musara	2016	85	correlational	NA	NA	Career success (career management and talent management effectiveness)	Self-developed items (no example given)	.66
Ollo-Lopez et al.	2010	4,251	correlational	Job rotation	Indicate common situation at workplace: change job position (= 1) or always at the same job position (= 0)	Job involvement (voluntary effort)	Usually, do you extend your working hours for personal reasons with or without overtime payment?	–.05
		12,056	correlational	Task rotation	Do you always perform the same tasks in your firm? (yes/no)	Job involvement (voluntary effort)	I am willing to work beyond what I should for the success of my organization.	.05
Petit et al.	2015	1532	correlational	both	Job/task rotation ≥ once per week (yes/no)	Musculoskeletal complaints (carpal tunnel syndrome)	Medical examination	.02

Authors	Year	*N*	Study rigor	Intervention	Operationalization	Outcome (original wording)	Operationalization	*r*
Pini & Santangelo	2005	199	correlational	NA	Firm practices job rotation (yes/no)	Innovativeness (product innovation)	Item, whether firm has introduced a product innovation	–.03
						Innovativeness (process innovation)	Item, whether firm has introduced a process innovation	.21
						Innovativeness (quality innovation)	Item, whether firm has introduced a quality innovation	.11
Roquelaure et al.	1997	130	correlational	Task rotation	Observation: number of rotations between different workstations	Musculoskeletal complaints (carpal tunnel syndrome)	Medical records	–.23
Roquelaure et al.	2009	3,275	correlational	Both terms	Job/task rotation ≥ once per week (yes/no)	Musculoskeletal complaints (upper-extremity musculoskeletal disorders)	Physical examination	.07
Sawhney	2013	74	correlational	Job rotation	Items based on previous studies (e.g., extent to which plant floor employees are trained by rotating between work centers)	Labor flexibility (labor flexibility acquired)	Proportion of operators able to perform multiple jobs	.57
						Labor flexibility (labor flexibility implemented)	Self-developed items (e.g., the extent to which the labor practices allowed workers to be used for multiple jobs)	.55
						Financial performance (plant performance)	Self-developed items (e.g., rating manufacturing cost compared to competitors)	.11
Selden et al.	2013	42	correlational	NA	Percentage of employees with access to job rotation: no employees, some employees (5–49%), most employees (50–99%), all employees	Turnover intention (voluntary turnover)	Company data	–.23
Shin	2009	1,106	correlational	Job rotation	Number of employees currently involved in job rotation (defined as a work design system that allows employees to rotate among different jobs)	Turnover intention (turnover)	Company data	.01
Song et al.	2006	277	correlational	Job rotation	Items from previous study (e.g., we rotate chairmanships of new product development among R&D, manufacturing, and marketing personnel)	Organizational commitment (individual commitment)	Items adapted from previous study (e.g., extent to which people defended the company when others criticize it)	–.16
						Financial performance (organizational crisis)	Items from previous studies (e.g., frequency of organizational crises)	.04
						Financial performance (relative costs)	Company data	.01

Authors	Year	*N*	Study rigor	Intervention	Operationalization	Outcome (original wording)	Operationalization	*r*
Song et al.	2010	146	correlational	Job rotation	Senior management promotes job rotation across departments (strongly agree – strongly agree)	Innovativeness (innovation performance)	From an overall profitability standpoint, our new product development program has been successful	.54
		185	correlational	Job rotation	Items from previous studies (e.g., we rotate chairmanships of new product development among R&D, manufacturing, and marketing personnel)	Innovativeness (innovation performance)	Self-developed items (e.g., Compared with our major competitors, our new product development program is far more successful)	–.11
Tsai & Huang	2020	4,381	correlational	Task rotation	NA	Innovativeness (significant innovation)	Company data: ratio of the firm's sales from front-stage innovation divided by its total sales	.10
						Innovativeness (incremental innovation)	Company data: ratio of the firm's sales from front-stage innovation divided by its total sales	–.12
						Financial performance (sales growth)	Company data: average growth rate of sales revenue	.06
Weichel et al.	2010	248	correlational	Task rotation	Objective company data: number of workstations that employees work on	General physical health (work ability)	Work Ability Index (Ilmarinen & Tempel, 2002)	.35
						General physical health (take days off)	Company data	.21
						General physical health (cases of illness)	Company data	.24
						Musculoskeletal complaints	Modified version of Body Part Discomfort Scale (Corlett & Bishop, 1976)	–.35
						Stress and burnout (cognitive and emotional strain)	Irritation scale (Mohr et al. 2005)	–.23
						Job involvement (resignation)	Work-related behaviour and coping styles inventory; (Schaarschmidt & Fischer, 1996)	.22
Zhu et al.	2013	47	correlational	Job rotation	Objective company data: number of job titles held by the person	Labor flexibility (adaptability)	Expert judgment whether employees could work in a variety of situations, and with various individuals or groups	–.41
Zwick	2002	2,460	correlational	NA	Job rotation offered in 1997? (yes/no)	Organizational performance (productivity)	Company data	.02
						Organizational performance (productivity)	Company data	.02

Note. [a]Study reported relationship for multiple groups, values indicate range.
[b]First value indicates comparison with low-intensity work, second value indicates comparison with high-intensity work
